# Synergistic antiviral effects of structure-guided peptides and a mutagenic base analog on SARS-CoV-2 replication

**DOI:** 10.1128/aac.01885-25

**Published:** 2026-04-27

**Authors:** Sergio Ortega Del Campo, Gregorio Joaquín Fernández Ballester, Clara Blanes Mira, Víctor Guirado Osorio, Luis Díaz Martínez, Ana Isabel de Ávila, María Eugenia Soria, Brenda Martínez-González, Francisco José Villena González, Josefa Gómez-Maldonado, María Isabel Viciana Ramos, Encarnación Clavijo Frutos, Jesús L. Santos González, Ugo Bastolla, Celia Perales, Esteban Domingo, Enrique Viguera, Ana María Fernández Escamilla, Ana Grande Pérez

**Affiliations:** 1 Área de Genética, Facultad de Ciencias, Campus de Teatinos, Universidad de Málaga 98702 , Málaga, Spain; 2 Instituto de Investigación en Biotecnología y Salud (IDiBE), Universidad Miguel Hernández, Elche, Spain; 3 Department of Public Health, History of Science and Gynecology, Universidad Miguel Hernández 552188, Sant Joan d'Alacant, Spain; 4 Centro de Supercomputación y Bioinnovación (SCBI), Universidad de Málaga 16752, Málaga, Spain; 5 Centro de Biología Molecular Severo Ochoa (CBM), (Consejo Superior de Investigaciones Científicas-Universidad Autónoma de Madrid) (CSIC-UAM), Campus de Cantoblanco, Madrid, Spain; 6 Department of Clinical Microbiology, Instituto de Investigación Sanitaria-Fundación Jiménez Díaz University Hospital, Universidad Autónoma de Madrid (IIS-FJD, UAM) 16722, Madrid, Spain; 7 Servicios Centrales de Apoyo a la Investigación (SCAI), Área de Ciencias de la Vida, Genómica, Universidad de Málaga 16752, Málaga, Spain; 8 Hospital Universitario Virgen de la Victoria, Campus de Teatinos 83159 , Málaga, Spain; 9 Instituto de Investigación Biomédica de Málaga y Plataforma en Nanomedicina (IBIMA Plataforma-BIONAND), Universidad de Málaga 16752, Málaga, Spain; Chinese Academy of Medical Sciences & Peking Union Medical College, Beijing, China

**Keywords:** SARS-CoV-2, ExoN activity, MTase activity, nsp10, 5-fluorouracil, peptide design, drug combination, synergism, mutant spectra, lethal mutagenesis

## Abstract

The limited durability of vaccine protection and the rapid emergence of SARS-CoV-2 variants highlight the need for antiviral strategies that extend beyond vaccination and conventional small-molecule inhibitors. Here, we explored a dual approach combining structure-guided peptides predicted to interfere with the viral replication complex with lethal mutagenesis to limit SARS-CoV-2 replication. Using the crystallographic interfaces of nsp10 with nsp14 and nsp16, we designed short inhibitory peptides predicted to interact with the viral proofreading and RNA-capping machinery. In parallel, the mutagenic analog 5-fluorouracil was evaluated to determine its effect on SARS-CoV-2 in Vero E6 cells. Peptides P1 and P6 exhibited potent antiviral activity with minimal cytotoxicity, whereas 5-FU reduced specific infectivity without impairing genome replication. Combined treatment with 5-FU and peptide P1 resulted in >10^4^-fold reduction in infectious virus, achieving near-complete loss of infectivity at non-cytotoxic concentrations. Next-generation sequencing revealed that dual treatment increased mutation frequency, altered mutant spectra, and decreased genome stability, consistent with progression toward error catastrophe. Principal component analysis confirmed that combined treatment generated mutant spectra distinct from either monotherapy. These findings are consistent with a dual antiviral strategy in which structure-guided peptides designed to interact with components of the SARS-CoV-2 replication complex act in combination with lethal mutagenesis to produce a synergistic interaction between these two complementary processes. This integrated approach suggests a potential broad-spectrum antiviral strategy with applicability to other coronaviruses.

## INTRODUCTION

Although vaccination remains a cornerstone for controlling the SARS-CoV-2 pandemic, the emergence of new variants has raised concerns about reduced vaccine effectiveness. Recent variants have shown lower sensitivity to vaccine-induced neutralization (1–6). The high mutation rate inherent to RNA virus replication gives rise to mutant swarms or viral quasispecies (7, 8).

A key consequence of quasispecies dynamics is the rapid emergence of drug- and antibody-resistant mutants (9, 10). These minority variants may harbor advantageous mutations, become dominant within viral populations, and ultimately undermine conventional antiviral strategies (11–14). Therefore, novel antiviral strategies are urgently needed to complement vaccination, particularly against emerging variants capable of immune escape.

Building on viral mutation dynamics and quasispecies theory, lethal mutagenesis has been proposed as an antiviral strategy aimed at driving viruses to extinction by increasing the replication error rate beyond a tolerable threshold, promoting the accumulation of deleterious mutations and loss of infectivity (15, 16). This principle has been validated using base and nucleoside analogs that enhance mutagenesis and cause viral extinction in several animal and plant RNA viruses (17–20). Among these compounds, the nucleoside analog β-D-*N*^4^-hydroxycytidine (NHC; molnupiravir) induces lethal mutagenesis in SARS-CoV-2 and effectively inhibits infection (21, 22). More recently, the synergistic combination of remdesivir and ribavirin, based on lethal mutagenesis, has been explored for SARS-CoV-2 inhibition (23).

In addition to increasing mutation rates, analogs can reshape viral genome composition, including dinucleotide usage patterns. RNA viruses evolve under compositional constraints that shape genome structure, including marked suppression of CpG and UpA dinucleotides (24, 25). Reduced CpG content is believed to facilitate evasion of the antiviral zinc-finger protein ZAP, which binds CpG dinucleotides and directs them toward degradation via cofactors such as KHNYN (26–28). SARS-CoV-2 exhibits extreme CpG deficiency, lower than that of other betacoronaviruses, suggesting strong selective pressure to evade ZAP- and APOBEC-mediated innate immune pathways (29–32).

However, the 3′→5′ exonuclease proofreading activity of coronaviruses (33) may counteract the mutagenic effects of nucleoside analogs (34). To overcome this barrier, antiviral peptides designed to target SARS-CoV-2 replication proteins have emerged as a promising complementary strategy (35–37). One particularly attractive target is nsp10, a multifunctional cofactor that interacts with nsp14 and nsp16, modulating both the exonuclease activity of nsp14 and the 2′-O-methyltransferase (2′-O-MTase) activity of nsp16 (38, 39). Nsp10 is highly conserved among coronaviruses due to its essential roles in replication fidelity and genome maintenance (40, 41). Consequently, the binding interfaces of the nsp10-nsp14 and nsp10-nsp16 complexes represent promising targets for structure-guided inhibitory peptides (42–46).

The objective of this study was to design and evaluate a combinatorial antiviral therapy that integrates mutagenic and peptide-based inhibitors against SARS-CoV-2. We selected 5-fluorouracil (5-FU) as the mutagen, based on computational predictions of its effects on the Wuhan-Hu-1 genome and prior evidence of its antiviral activity in other RNA viruses (18, 20, 47). For peptide design, we used crystal structures of SARS-CoV and SARS-CoV-2 to identify the nsp10-nsp16 and nsp10-nsp14 interfaces and selected peptide fragments based on length, secondary structure, and predicted binding energy. Peptides were ranked according to interaction energy and intramolecular stability and were predicted to interact with nsp14 and nsp16, potentially interfering with the formation of functional heterodimers required for viral replication (42, 43, 46). We then evaluated their cytotoxicity and antiviral efficacy, individually and in combination with 5-FU, in Vero E6 cells infected with the Omicron BA.1.17 variant. Viral infectivity was quantified by plaque assay, and viral RNA, by RT-qPCR. Selected samples were subsequently analyzed by next-generation sequencing (NGS) to characterize mutant spectra and genetic diversity.

## MATERIALS AND METHODS

### Computational prediction of the effect of lethal mutagenesis on SARS-CoV-2

A computational framework was used to predict the effect of different types of base substitutions on SARS-CoV-2 replicative fitness. These analyses simulated the potential action of nucleoside analogs by evaluating their expected impact on dinucleotide frequencies and protein stability.

### Estimation of dinucleotide effects

The contribution of each dinucleotide to viral fitness was estimated by comparing its observed frequency in the viral genome with the expected frequency based on overall nucleotide composition, using the following formula:


$$Fitness\;(XY)=log\left(\frac{freq_{observed}\;(XY)}{freq_{expected}\;(XY)}\right)$$

Calculations were performed using the Wuhan-Hu-1 reference genome (GenBank accession NC_045512.2). The estimated impact of mutations on fitness was derived from the expected effect of each substitution type on dinucleotide frequency. This analysis is inherently linked to both the number and distribution of mutations and dinucleotides along the genome.

### Estimation of mutational costs

The effects of amino acid substitutions on protein stability were predicted as a proxy for fitness using the DeltaGREM method (48). This model estimates the protein folding free energy, ΔG, as the difference between the free energy of the native state, estimated through the contact free energy parameters developed in a previous study (49), and the free energy of compact misfolded conformations, estimated using a contact-based model developed (50) in analogy with the Random Energy Model (51). Fitness was estimated from these values using the following equation:


$$Fitness=\frac{\exp\left(\frac{-\;\Delta G}{RT}\right)}{1\;+\;\exp\left(\frac{-\;\Delta G}{RT}\right)}$$

For all possible amino acid changes in each viral protein, DeltaGREM calculates the corresponding change in folding free energy ΔΔG. The program was applied as described by de la Higuera et al. (52) to estimate the mutational load produced by different mutational spectra.

We analyzed all SARS-CoV-2 proteins with experimentally resolved structures in the Protein Data Bank (PDB) together with their corresponding nucleotide sequences. For each protein, we predicted the average value of ΔΔG for all possible mutations at the nucleotide level and evaluated the corresponding changes at the amino acid level. This enabled the prediction of the structural and stability impact of every type of mutation at the nucleotide level.

### Computational peptide design

#### Visualization and identification of interacting regions

Crystal structures of SARS-CoV-2 nsp10-nsp14 (PDB 7DIY) and nsp10-nsp16 (PDB 6W4H) complexes were retrieved from the RCSB PDB (https://www.rcsb.org/). Contact regions were identified by visual inspection using PyMOL v3 (The PyMOL Molecular Graphics System, Version 3.0 Schrödinger, LLC; https://www.pymol.org/). Nsp10 was fragmented into 9–12 amino acid segments used as ligand candidates, while nsp14 or nsp16 served as receptors for computational modeling. The resulting complexes were isolated for sequence optimization and energy evaluation.

A conservation analysis of the interaction interfaces was also performed. Protein sequences of nsp10, nsp14, and nsp16 from representative coronaviruses were retrieved, and genomic regions were extracted from annotated reference genomes. Multiple sequence alignments were generated with MAFFT using default parameters (53). Contact residues of the nsp10-nsp14 and nsp10-nsp16 complexes were then analyzed, and sequence logos visualizing positional conservation and variability were produced using WebLogo (https://weblogo.threeplusone.com/create.cgi).

#### Interaction energy calculations

Interaction energies were computed using FoldX v5.0 (https://foldxsuite.crg.eu/), which estimates contributions from electrostatic interactions, hydrogen bonds, disulfide bridges, van der Waals forces, dipole interactions, solvation, and entropic factors. The sum of these contributions yielded total estimated interaction energy, and complexes with the most favorable (most negative) values were selected for further optimization.

**Fig 1:**
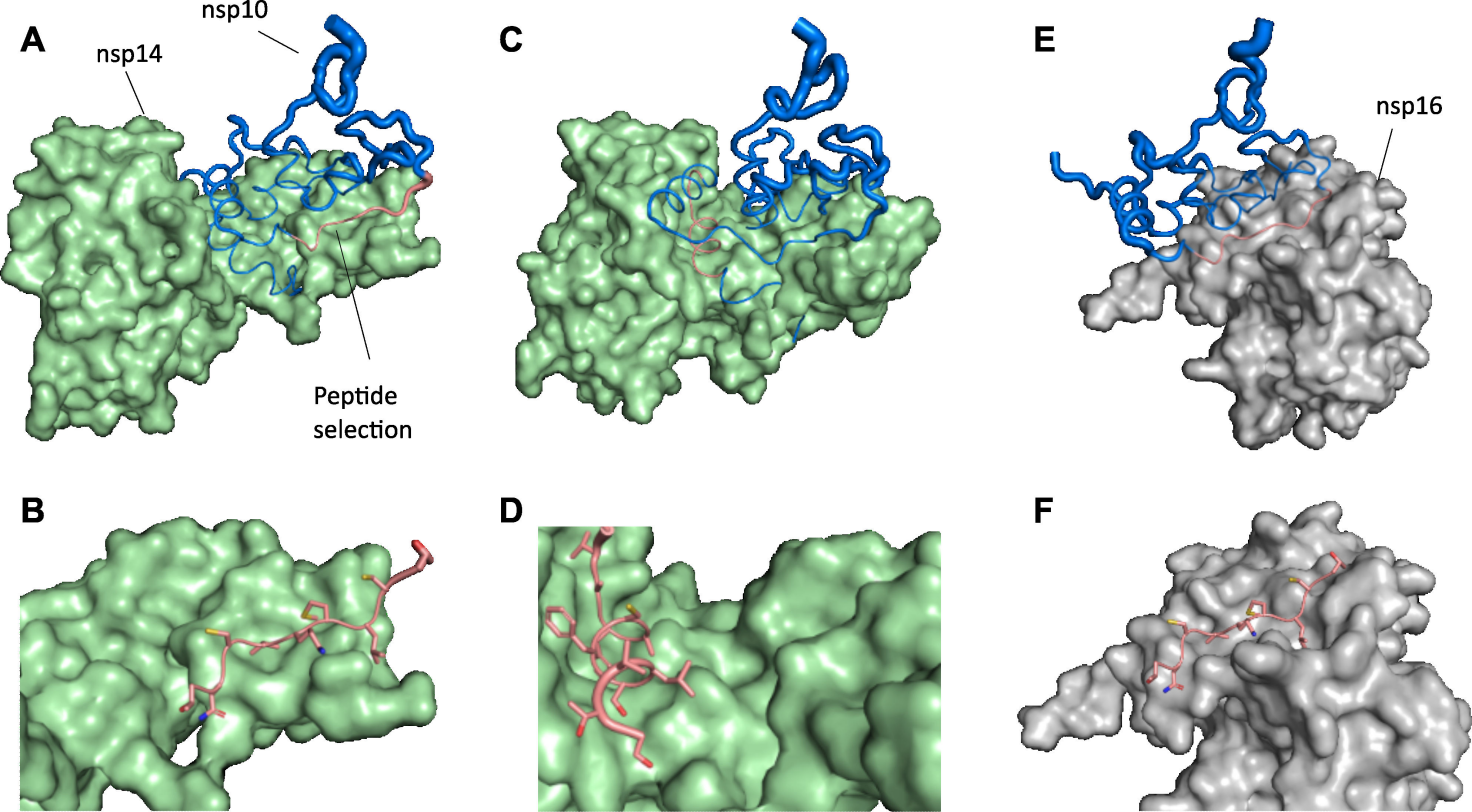
Structural basis for the design of inhibitory peptides designed to target the SARS-CoV-2 nsp10-nsp14 and nsp10-nsp16 interfaces, based on computational modeling. (**A**) Interface of nsp14 exoribonuclease (green surface) contacting nsp10 (blue cartoon). The nsp10 segment 39-TNCVKMLCT-47 used as a template for peptide design is shown in salmon. (**B**) Close-up of the nsp14-peptide complex after deleting the remaining nsp10 residues, showing the region used for binding-energy calculation. (**C**) Alternative contact region in nsp10 corresponding to helix α1 (11-STVLSFCAFAVD-22; salmon), considered an additional nsp14-binding motif. (**D**) Structural context of helix α1 within nsp10, highlighting its orientation and interaction surface. (**E**) Interaction between nsp16 O-methyltransferase (gray surface) and nsp10 (blue), showing that the same 39-TNCVKMLCT-47 region (salmon) contributes to binding. (**F**) Close-up of the nsp16-peptide complex used for energy scoring. Structures were derived from the SARS-CoV-2 nsp14 ExonN complex (54) and the nsp16 O-MTase complex (55). These structural elements were used as input for computational design and optimization of candidate inhibitory peptides.

For the nsp10-nsp14 interface, the nsp10 peptide 39-TNCVKMLCT-47 (9 aa) was chosen as the ligand, and the full-length nsp14 was used as the receptor (Fig. 1A and B). The same region was later considered within the nsp10-nsp16 complex to evaluate its potential dual-target interaction *in silico* (Fig. 1E and F). An additional contact region within nsp10, corresponding to helix α1 (11–STVLSFCAFAVD-22), was also considered an alternative binding motif toward nsp14 (Fig. 1C and D).

#### Selection of optimal peptide sequences

Bound nsp10 peptides were optimized in FoldX by considering the local amino acid environment (56). Position-specific scoring matrices were generated and visualized as color-coded maps to identify residues contributing most to binding affinity (Fig. S1).

Two iterative rounds of sequence optimization were performed, combining the best-scoring residues per position. Positions were classified as buried, semi-buried, or exposed, generating 1,000–5,000 variants per round and ranked by predicted binding energy. Round 1 prioritized buried and semi-buried positions, typically improving ΔGbinding by ≈ 3 kcal/mol relative to the wild-type fragment. Round 2 refined exposed and key positions and produced an additional ≈ 1 kcal/mol improvement on average.

Selection criteria included predicted interaction energy, hydrophobic content, and sequence similarity to the native fragment. The top eight optimized peptides (9–12 aa) were selected for *in vitro* assays. To improve intracellular stability, N-terminal acetylation and C-terminal amidation were introduced.

### Cell culture assays

All experiments involving infectious SARS-CoV-2 were conducted in a BSL-3 facility at the Centro de Biología Molecular Severo Ochoa (CBM).

#### Cells and virus

Vero E6 cells (ATCC Cat# CRL-1586, RRID:CVCL_0574) were cultured in DMEM (Merck, Darmstadt, Germany) supplemented with 10% fetal bovine serum (Sigma, St. Louis, USA), 50 μg/mL gentamicin (Pan Biotech, Aidenbach, Germany), 4 mM L-glutamine (Merck), 0.2 μg/mL antifungal (Sigma), and 1% non-essential amino acids (Merck). Cell growth and maintenance followed established protocols (23, 57).

A nasopharyngeal swab from a severe COVID-19 patient (Hospital Clínico Virgen de la Victoria, Málaga) was used for virus isolation. Viral stocks were generated by infecting 3 × 10^6^ Vero E6 cells at an MOI of 0.001 PFU/cell for 48 h at 37°C.

#### Drug exposure and cytotoxicity assays

Peptides were dissolved at 5 mg/mL in DMSO and stored at −80°C. 5-FU (Sigma, St Louis, USA) was prepared as a 3 mM stock solution in DMEM. Peptide cytotoxicity was assessed using a concentration-response design, testing multiple concentrations up to a maximum of 70 μM, whereas 5-FU was evaluated over a wider concentration range. Cytotoxicity was assessed in 96-well plates (10^4^ cells/well) after 72 h of exposure. MTT was added for 4 h, followed by 100 μL DMSO to dissolve the formazan crystals, and absorbance at 550 nm was measured to determine 50% cytotoxic concentration (CC_50_); when no 50% reduction in cell viability was observed within the tested peptide concentration range, CC_50_ values are reported as >70 µM.

#### Antiviral efficacy assays

Antiviral activity was assessed in six-well plates using virus passage 3. After 2 h of adsorption at 37°C, the inoculum was removed and replaced with a drug-containing medium. Infected cultures were harvested after 48 h.

Infectious titers (PFU/mL) were obtained by plaque assay, and IC_50_ and therapeutic index (TI = CC_50_/IC_50_) were calculated.

Additionally, peptide internalization assays were performed in cells to evaluate the cellular uptake of peptides P1 and P7. These two peptides were selected as representative active candidates for assessing intracellular uptake. Vero E6 cells (6.25 × 10^5^ per well) were seeded in six-well plates under standard culture conditions. GFP-labeled P1 and P7 were prepared from 5 mg/mL DMSO stocks and added directly to the cultures at final concentrations of 0.7, 7, and 70 μM. Cells were collected at 0, 6, 24, 36, and 48 h after peptide addition and examined by fluorescence microscopy. Three independent replicate wells were processed for each concentration and time point to ensure reproducibility and minimize potential phototoxicity during imaging.

Drug interactions were analyzed using the Chou-Talalay method with *CompuSyn* software, which computes combination indices (CI) to classify interactions as synergistic (CI < 1), additive (CI = 1), or antagonistic (CI > 1).

### Characterization of mutant spectra

#### RNA extraction, amplification, and NGS

Supernatants from selected cultures (Table S1A) were processed for sequencing. Viral RNA was extracted using the QIAamp Viral RNA Mini Kit (Qiagen, Germany). Twelve overlapping ~3 kb amplicons covering the full SARS-CoV-2 genome were generated by RT-PCR using the Transcriptor One-Step RT-PCR Kit (Roche, Germany) (Table S1B). Amplicons were gel-purified with the QIAquick Gel Extraction Kit (Qiagen) and sequenced at the SCAI Ultrasequencing Unit (University of Málaga). Libraries were prepared with the Nextera XT DNA Library Prep kit (Illumina) and sequenced on an Illumina NextSeq 550 platform (2 × 150 bp). Standard Illumina pipelines were used for base-calling and demultiplexing.

In parallel, the passage-3 viral stock used in the experiments was sequenced using the COVID-Seq protocol (Illumina), allowing variant assignment (Omicron variant, Pango BA.1.17, clade 21K) and the identification of characteristic mutations relative to the Wuhan-Hu-1 reference genome (NC_045512.2) (Fig. S2). The sequence has been deposited in GenBank under accession number PV483427.1.

#### Viral RNA quantification

Viral RNA was quantified by RT-qPCR using the LightCycler RNA Master SYBR Green Kit and LightCycler II system (Roche, Switzerland). Genome copy numbers were calculated from a standard curve generated with *in vitro*-transcribed RNA from the WA1/2020 SARS-CoV-2 strain. Specific infectivity (SI) was calculated as the ratio of infectious units (PFU) to total RNA genomes.

#### Bioinformatic analysis

Fastq files were processed following established pipelines (58, 59). Quality filtering was performed with FastQC and fastp (60). Clean reads were aligned to the SARS-CoV-2 reference genome PV483427.1 using BWA-MEM and processed with SAMtools. Minority variants were identified from each sample’s consensus-aligned reads using LoFreq (frequency cutoff ≥0.1%) (61).

Genetic diversity metrics included mutation frequency, nucleotide diversity, and Shannon entropy. Statistical tests included Shapiro-Wilk for normality and Levene’s test for variance homogeneity. Depending on the distribution, one-way ANOVA or Kruskal-Wallis tests were applied.

Mutation randomness was evaluated genome-wide and per ORF using a non-parametric runs test (*P* < 0.05). Dinucleotide frequencies were computed to assess compositional shifts in the mutant spectra.

## RESULTS

### Computational prediction of the effect of lethal mutagenesis on SARS-CoV-2

**Fig 2:**
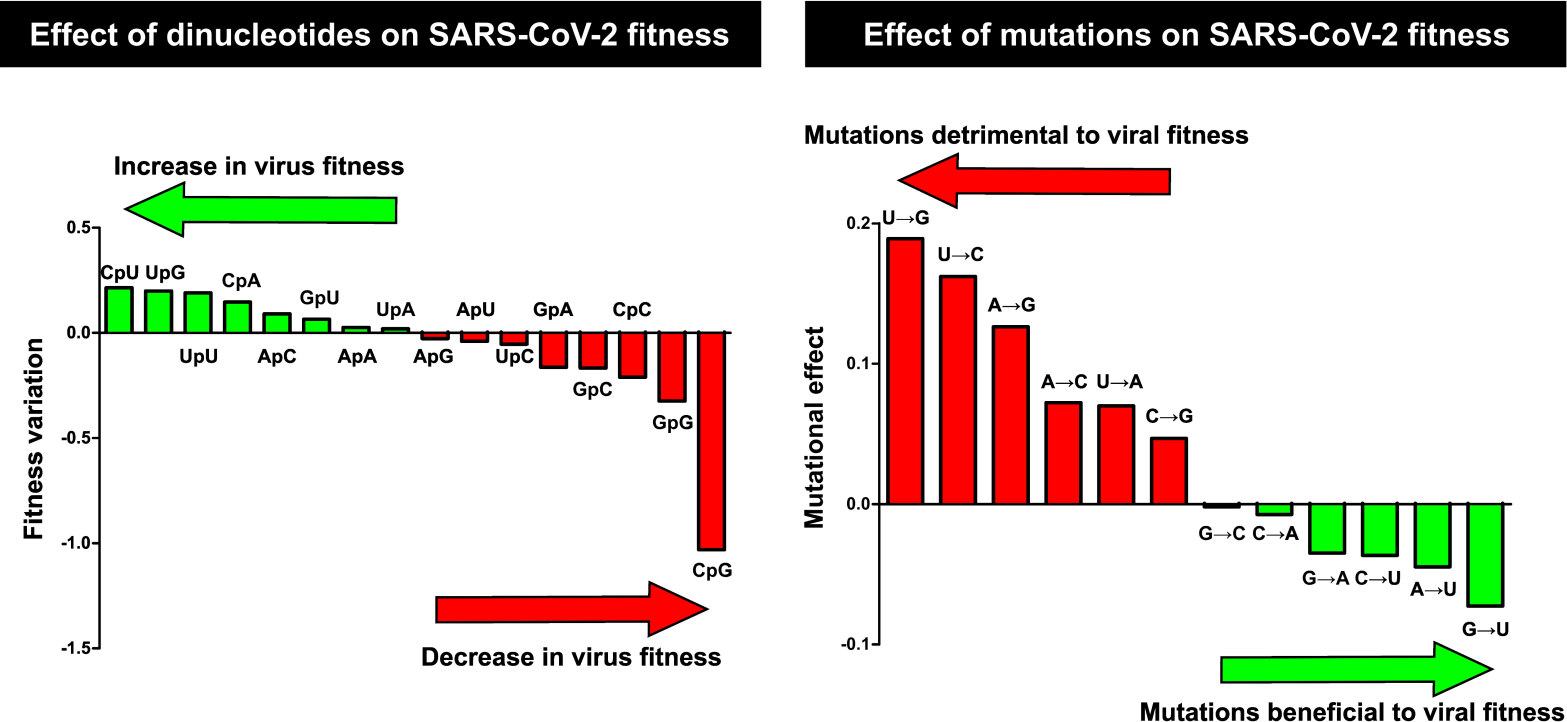
Left: Estimated effects of dinucleotides on SARS-CoV-2 fitness. Negative values indicate that the presence of these dinucleotides confers a fitness disadvantage, whereas positive values indicate a beneficial effect. Right: Predicted fitness effects of each mutation based on its expected impact on dinucleotide composition. Positive values indicate that the mutation increases unfavorable dinucleotides and/or reduces favorable dinucleotides. Negative values indicate the opposite pattern. Wuhan-Hu-1 (NC_045512.2) was used as the reference.

Dinucleotides with the lowest fitness scores were enriched in cytosine and guanine, particularly CpG, whose negative values indicated a detrimental impact on SARS-CoV-2 fitness. In contrast, CpU, UpG, and UpU displayed positive fitness values, suggesting that these dinucleotides favor viral replication. Mutational-load analysis revealed strong predicted fitness penalties for substitutions that increase disfavored dinucleotides. Among all mutation types, U→G, U→C, and A→G produced the highest mutational loads (Fig. 2), consistent with their tendency to increase G/C content.

When evaluating the fitness cost of each mutation across individual viral proteins, the most deleterious substitutions were U→G, U→A, and U→C (Table 1; Table S2). The predicted burden varied depending on protein identity and secondary structure. For example, in nsp10, U→G and U→A were most damaging, whereas in heterodimeric complexes (nsp10-nsp14 and nsp10-nsp16), as well as spike, G→U and C→U produced the strongest destabilizing effects.

**TABLE 1:** Effect of each substitution type on the fitness of SARS-CoV-2 proteins

Cost of mutations in the SARS-CoV-2 proteins		
Mutation^*a*^	nmut^*b*^	Mutation load^*c*^
U→G	5,194	0.0574
U→A	5,194	0.0544
U→C	5,194	0.0459
G→U	3,396	0.0451
A→U	4,963	0.0445
G→C	3,396	0.0311
C→U	3,214	0.0248
A→C	4,963	0.0210
G→A	3,396	0.0209
C→A	3,214	0.0148
C→G	3,214	0.0134
A→G	4,963	0.0075

^*a*^ The mutations shown in the table are sorted according to their mutation load value in descending order.

^*b*^ nmut is the total number of nucleotide mutations.

^*c*^ Mutation load = Total estimated fitness cost of mutations, measured by ΔΔG or change in the free energy of folding of a protein caused by a mutation; indicates whether the mutation stabilizes (ΔΔG < 0) or destabilizes (ΔΔG > 0) the protein.

Our results indicate that mutations that change uracil to any other nucleotide are especially detrimental for viral fitness. Mutations from other nucleotides to U, which increase hydrophobicity, are also detrimental, but less so. These computational results supported the selection of 5-FU, which predominantly induces U→C and A→G transitions (18, 62, 63), as a suitable mutagenic analog for experimental testing. Although not previously evaluated in SARS-CoV-2, 5-FU displays mutagenic activity in SARS-CoV (34, 45).

### Computational peptide design

Using the structural templates defined in Methods (Fig. 1), two regions of nsp10, residues 39–47 and helix α1 (positions 11–22), were optimized as scaffolds for peptide inhibitors designed to interact with the nsp14 and nsp16 interfaces. These segments correspond to the principal interaction interfaces observed in crystallographic complexes.

For the nsp10-nsp14 interface, the native fragment 39-TNCVKMLCT-47 exhibited a predicted interaction energy of –9.63 kcal/mol. FoldX optimization produced peptide 39-QLQIYLMRK-47, with improved predicted interaction energy (–12.99 kcal/mol) and reduced hydrophobic content, yielding a net charge of +2 compatible with the nsp14 electrostatic environment.

The α1 helix region (11-STVLSFCAFAVD-22) displayed a higher predicted intrinsic interaction energy (–15.45 kcal/mol). Two rounds of guided mutagenesis generated the optimized sequence 11-KIEKTFEYYMVR-22, with substantially improved predicted interaction energy (–26.27 kcal/mol). Phe16 and Val21 were retained due to their buried hydrophobic roles, consistent with their conservation across coronaviruses.

For nsp10-nsp16, optimization of the same 39–47 region yielded peptide 39-ELKIRMRWK-47, improving predicted interaction energy from –12.17 kcal/mol to –16.7 kcal/mol, driven by increased positive residues consistent with the nsp16 electrostatic landscape.

In total, eight peptides (9–12 aa) were selected for synthesis and biological evaluation (Table 2) based on (i) predicted interaction energy, (ii) physicochemical suitability, and (iii) complementarity to conserved interaction surfaces (Fig. 1; Fig. S1).

**TABLE 2:** Peptides designed on SARS-CoV-2 nsp10 and selected for *in vitro* assays

Peptide	Sequence	MW^*a*^	Net charge	Identity with WT (%)	Predicted interaction energy (kcal/mol)	Target protein^*b*^
P1	ELKIRMRWK	1,300.63	+3	1 (11%)	−16.70	nsp16
P2	ELKILMMWK	1,232.61	+1	1 (11%)	−16.97	nsp16
P3	QLQILMMKK	1,173.54	+2	1 (11%)	−13.03	nsp14
P4	ELKILLMKK	1,156.53	+2	0 (0%)	−12.89	nsp14
P5	QLQIIMMRK	1,201.56	+2	1 (11%)	−13.05	nsp14
P6	QLQIYLMRK	1,233.53	+2	0 (0%)	−12.99	nsp14
P7	KIEKTFEYYMVR	1,647.94	+1	2 (17%)	−26.27	nsp14
P8	KIERTFQYYMVR	1,674.97	+2	2 (17%)	−26.23	nsp14

^*a*^ Molecular weight.

^*b*^ Peptides P1–P6 were derived from the nsp10 region spanning residues 39–47 (TNCVKMLCT), which participate in both nsp10-nsp14 and nsp10-nsp16 interfaces. Although individually optimized toward one target based on structural modeling, potential cross-interaction with either protein cannot be excluded. Peptides P7 and P8 were derived from the helix α1 region of nsp10 (residues 11–22: STVLSFCAFAVD), primarily involved in the nsp10-nsp14 interaction.

**Fig 3:**
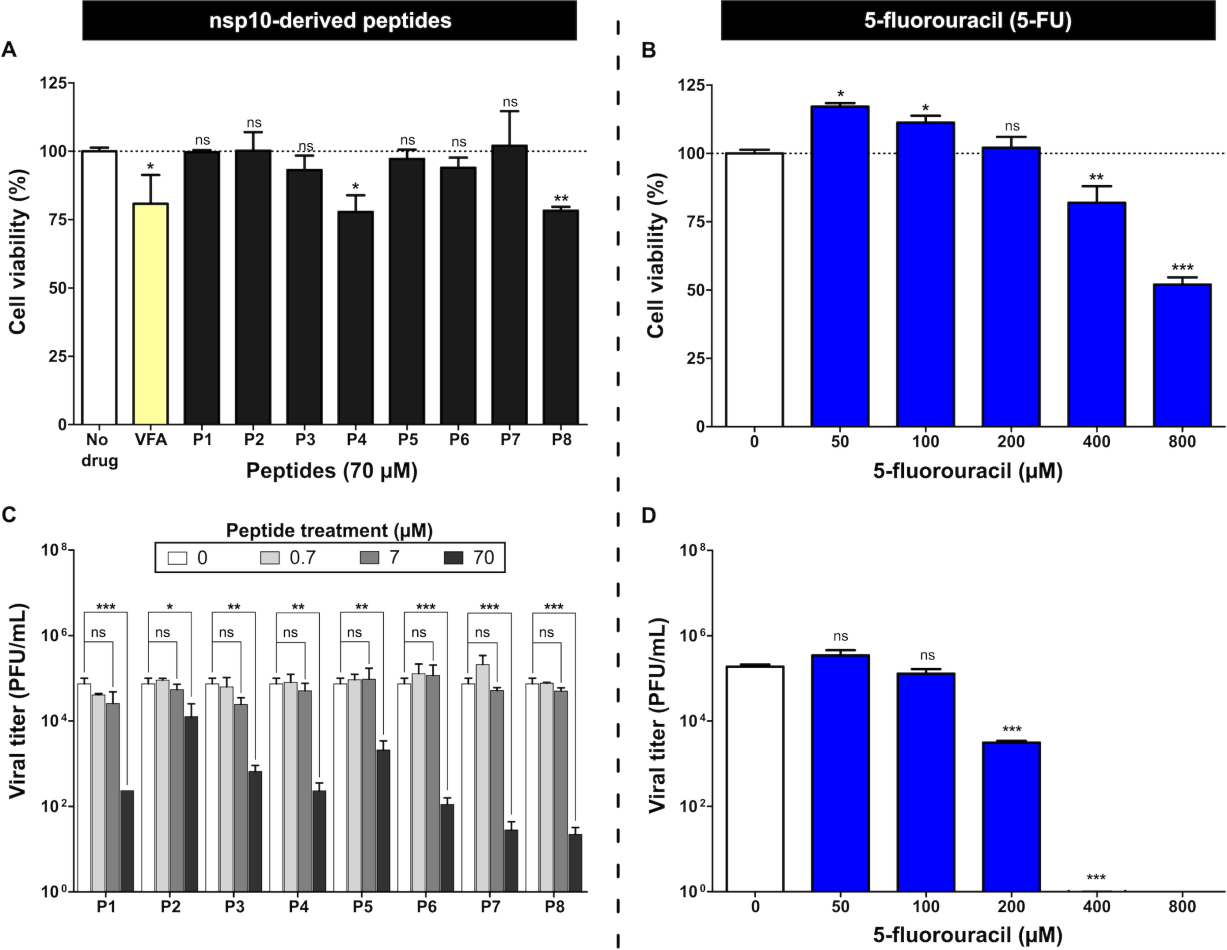
Initial screening of cytotoxicity and antiviral activity of peptides and 5-FU in Vero E6 cells. Cell viability (%) was determined by MTT assay after 72 h of exposure. Cytotoxicity was evaluated using a concentration-response design. (**A**) Cells treated with peptides P1–P8 at the highest concentration tested (70 µM). A peptide targeting foot-and-mouth disease virus (VFA) was used as a control (Fig. S4). (**B**) Cells were treated with increasing concentrations of 5-FU. Complete concentration-response absorbance data and calculated percentages of viability and mortality are summarized in Table S4A. Viral infectivity was quantified by plaque assay (PFU/mL) 48 h post-infection. Dose-response analysis was used to identify peptides showing antiviral activity within the non-cytotoxic concentration range tested. (**C**) Cultures treated with peptides P1–P8 at increasing concentrations (0.7–70 µM) to evaluate their effects on viral infectivity. (**D**) Cultures were treated with 5-FU at increasing concentrations. Complete viral titer data are summarized in Table S4B. Bars represent mean ± SD (*n* = 3). Statistical analysis was performed by comparing each drug-treated condition with the untreated control. Asterisks indicate significant differences compared with the control (*P < 0.05; **P < 0.01; ***P < 0.001); ns, not significant.

Comparative alignments of nsp10, nsp14, and nsp16 across the *Orthocoronavirinae* revealed strong conservation in the targeted interaction regions (Fig. 3). Conservation was highest in alpha- and beta-coronaviruses, moderate in gamma-coronaviruses, and lower in delta-coronaviruses (Table S3A through D), suggesting that these peptides may potentially interact with equivalent interfaces across genera, which is consistent with a potential pan-coronavirus applicability.

**TABLE 3:** Cytotoxic (CC_50_), inhibitory (IC_50_, IC_75_), and therapeutic index (TI) values for 5-FU and nsp10-derived peptides

Compound	CC_50_^*a*^ (µM)	IC_50_^*b*^ (µM)	IC_75_ (µM)	TI (CC_50_/IC_50_)
5-FU	722.77	126.86	142.89	5.70
P1	> 70	4.50	12.10	> 15.56
P2	> 70	≥70 µM	154.87	> 0.64
P3	> 70	15.81	20.91	> 4.42
P4	> 70	41.89	54.57	> 1.67
P5	> 70	≥70 µM	87.96	> 1.07
P6	> 70	18.68	47.82	> 3.75
P7	> 70	17.07	38.21	> 4.10
P8	> 70	29.12	36.85	> 2.40

^*a*^ CC_50_ values are reported as >70 µM when no 50% cytotoxicity was reached within the tested peptide concentration range.

^*b*^ IC_50_ values that did not reach 50% inhibition within the tested concentration range (70 µM) should be interpreted as approximate estimates.

### Culture-based experiments

#### Individual cytotoxicity and antiviral activity of peptides and 5-FU

Cytotoxicity assays in Vero E6 cells, performed using a concentration-response design, indicated that none of the peptides reached 50% cytotoxicity within the tested concentration range (up to 70 µM), except P4 and P8, which caused mild cytotoxicity at the highest concentration tested (Fig. 3A; Table S4A). 5-FU showed minimal toxicity up to 100 µM, but concentrations ≥400 µM reduced viability, reaching <50% at 800 µM (Fig. 3B). The CC_50_ = 722.77 µM confirmed low cytotoxicity (Table 3).

Antiviral assays showed that several peptides reduced viral titers at 70 µM (Fig. 3C). The dose-response experiments were performed as an initial screening step to identify peptides with reproducible antiviral activity within the non-cytotoxic concentration range tested. The strongest inhibitor was P1 (IC_50_ = 4.50 µM; TI > 15). P3, P6, and P7 also showed measurable antiviral effects (Table 3). Although peptide P1 showed the clearest dose-dependent antiviral trend, the responses of P6 and P7 were less pronounced within the concentration range tested (Fig. S4). For peptides whose inhibitory activity did not reach 50% within the tested concentration range (up to 70 µM), IC_50_ values are therefore reported as ≥70 µM and should be interpreted as approximate estimates. P4 and P8 were excluded due to cytotoxicity.

Based on these screening results, peptides P1, P6, and P7 were prioritized for further evaluation. Selection criteria included (i) reproducible reductions in viral infectivity across independent experiments, (ii) absence of detectable cytotoxicity within the tested concentration range, (iii) representation of peptides derived from distinct nsp10 interaction regions, and (iv) potential complementarity with the mutagenic mechanism of 5-FU. P1 and P6 were selected for the main combination studies with 5-FU because they produced the most consistent antiviral reductions within the non-cytotoxic concentration range tested. P1 and P7 were used for cellular internalization assays, and P7 was additionally evaluated in exploratory low-dose combination experiments described below. This strategy allowed us to examine peptides with distinct antiviral behaviors while focusing the main combination analyses on the most consistent candidates.

Peptide internalization assays using GFP-conjugated P1 and P7 showed time- and concentration-dependent fluorescence signals consistent with cellular association and uptake. Fluorescence became detectable at 6 h for cultures treated with 7 μM and 70 μM, with P1 producing stronger and more homogeneous signals. Cell-associated fluorescence was broadly distributed throughout the cell area, with no granular or perinuclear pattern (Fig. S5). P7-treated cells showed weaker and more heterogeneous fluorescence, with maximal intensity at 24 h. At 0.7 μM, fluorescence was faint or absent for both peptides. No signal was detected in untreated control cells, and autofluorescence was negligible. Fluorescence intensity decreased by 36 h, particularly in P7-treated cultures. These observations indicate that both peptides, particularly P1, are capable of associating with cells and generating intracellular fluorescence signals in a time- and concentration-dependent manner.

In addition, the antiviral activity of 5-FU on SARS-CoV-2 was evaluated. Treatment with 5-FU alone produced no inhibition at ≤100 µM but caused a significant reduction at 200 µM and nearly complete loss of infectivity at ≥400 µM (Fig. 3D). The IC_50_ = 126.86 µM, well below the CC_50_ indicated effective inhibition of SARS-CoV-2 infectivity (Table 3).

#### Evaluation of drug combinations

**Fig 4:**
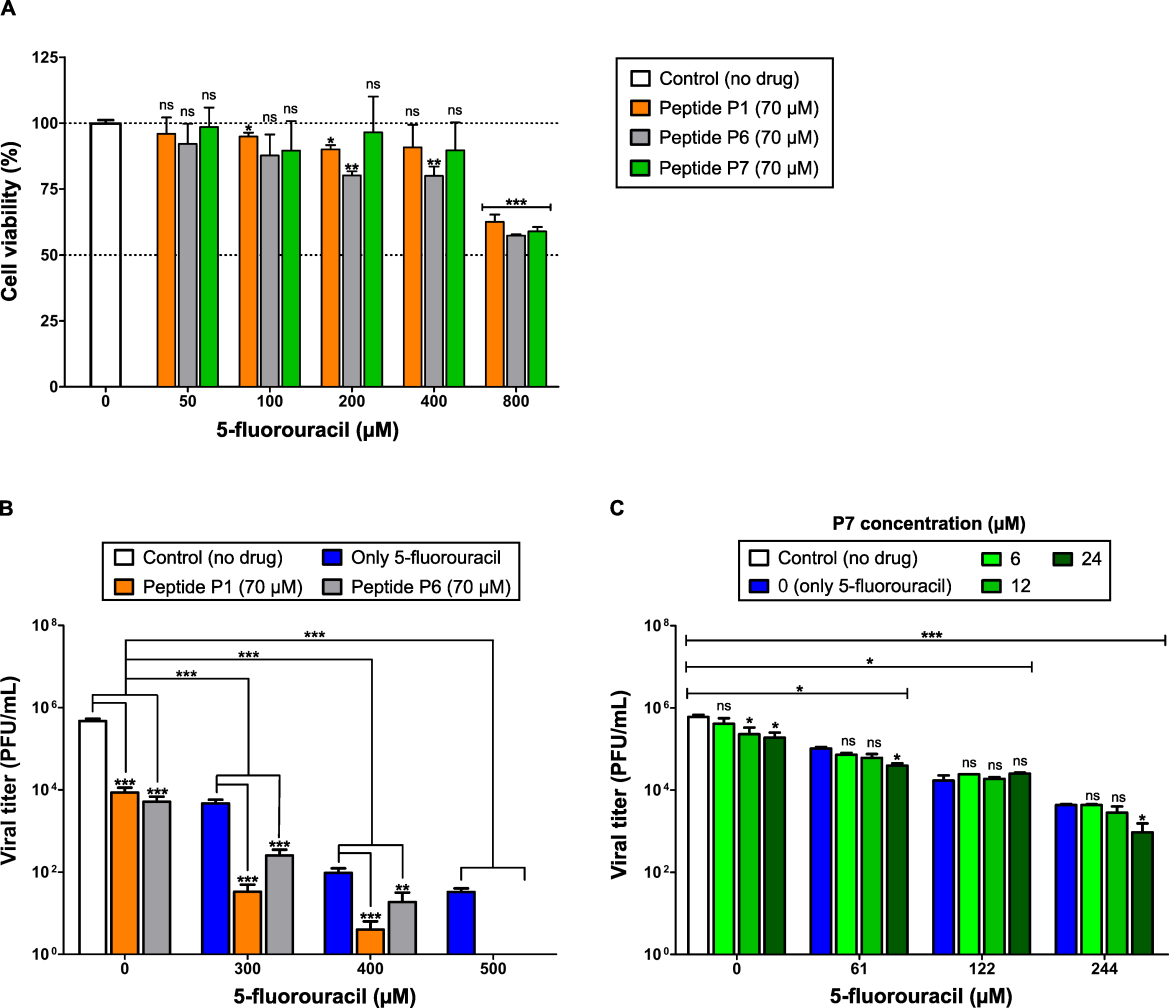
Screening and antiviral evaluation of peptide-5-fluorouracil combinations in Vero E6 cells. Cell viability (%) was measured by MTT assay after 72 h of exposure. Viral infectivity was quantified by plaque assay (PFU/mL) at 48 h post-infection. (**A**) Initial cytotoxicity screening of peptide-5-FU combinations. Vero E6 cells were treated with increasing concentrations of 5-FU in combination with peptides P1, P6, or P7 (70 µM). Cell viability data are summarized in Table S4A. (**B**) Antiviral activity of selected peptide-5-FU combinations. Cultures were treated with increasing concentrations of 5-FU in combination with peptides P1 or P6 (70 µM), and viral titers were determined by plaque assay. Viral titer values are summarized in Table S4B. (**C**) Exploratory low-dose antiviral evaluation of peptide P7 in combination with 5-FU. Cultures were treated with graded doses of 5-FU (61–244 µM) in combination with P7 (6–24 µM) to assess antiviral effects near the IC_50_ range of both compounds. Viral titer values are summarized in Table S4B. Bars represent mean ± SD (*n* = 3). For infectivity panels, two statistical comparisons were performed: (i) within each 5-FU concentration group, samples without peptide vs. samples treated with peptide; and (ii) between 5-FU-treated groups and the untreated control (X-axis = 0). White bars represent untreated controls (no drug). Statistical significance: *P < 0.05; **P < 0.01; ***P < 0.001; ns, not significant.

We next examined whether peptides enhanced the antiviral activity of 5-FU. To evaluate the cytotoxicity and antiviral effects of peptide-5-FU combinations, peptides selected from the initial screening (P1, P6, and P7) were evaluated in combination with 5-FU, based on their reproducible antiviral activity and absence of cytotoxicity within the tested concentration range. Peptides P1, P6, and P7 (70 µM) were tested with increasing concentrations of 5-FU to assess whether combination treatment could enhance antiviral activity or modify cytotoxic profiles. Among these peptides, P7 showed the lowest combined cytotoxicity (Fig. 4A).

Based on the screening assays, peptides P1 and P6 were selected for the main antiviral evaluation in combination with 5-FU because they showed the most consistent antiviral reductions across the tested concentration range, whereas P7 was examined separately in a low-dose exploratory analysis. P1 and P6 were evaluated across the full concentration range of 5-FU tested in this study, whereas P7 was examined at lower doses to determine whether partial enhancement of antiviral activity could be detected near the IC_50_ range of both compounds. The combination of 5-FU with peptides P1 or P6 significantly reduced viral titers beyond the corresponding monotherapies. Notably, the 5-FU + P1 combination consistently yielded the strongest inhibition. At 500 µM 5-FU, no detectable infectivity remained when either peptide was present, indicating near-complete loss of infectivity (Fig. 4B).

To explore whether partial antiviral enhancement could be observed at lower concentrations, P7 was tested in combination with 5-FU using graded doses near the IC_50_ of each compound. Virus titers decreased progressively with increasing 5-FU concentrations; however, adding P7 did not produce a significant additional inhibitory effect except at 24 µM peptide (Fig. 4C).

Chou-Talalay analysis revealed robust synergy for 5-FU + P1 across all concentrations, with CI values ranging from 0.471 to 0.932 (CI < 1 indicates synergy) (Fig. S6). In contrast, 5-FU + P6 showed an antagonistic effect at low doses (300 µM) but became synergistic at high 5-FU concentrations. Drug interaction analysis for 5-FU + P7 revealed a general trend toward antagonism, with weak synergy (CI = 0.831) only at the lowest concentrations tested (61 µM 5-FU and 6 µM P7).

### Effect of antivirals on the mutant spectra

Based on the culture results, we selected 5-FU and peptides P1 and P6 for a more detailed characterization of their antiviral effects. These conditions were chosen to evaluate how treatments modulated SARS-CoV-2 mutant spectra from RNA extracted from treated cultures.

#### Specific infectivity of SARS-CoV-2

**Fig 5:**
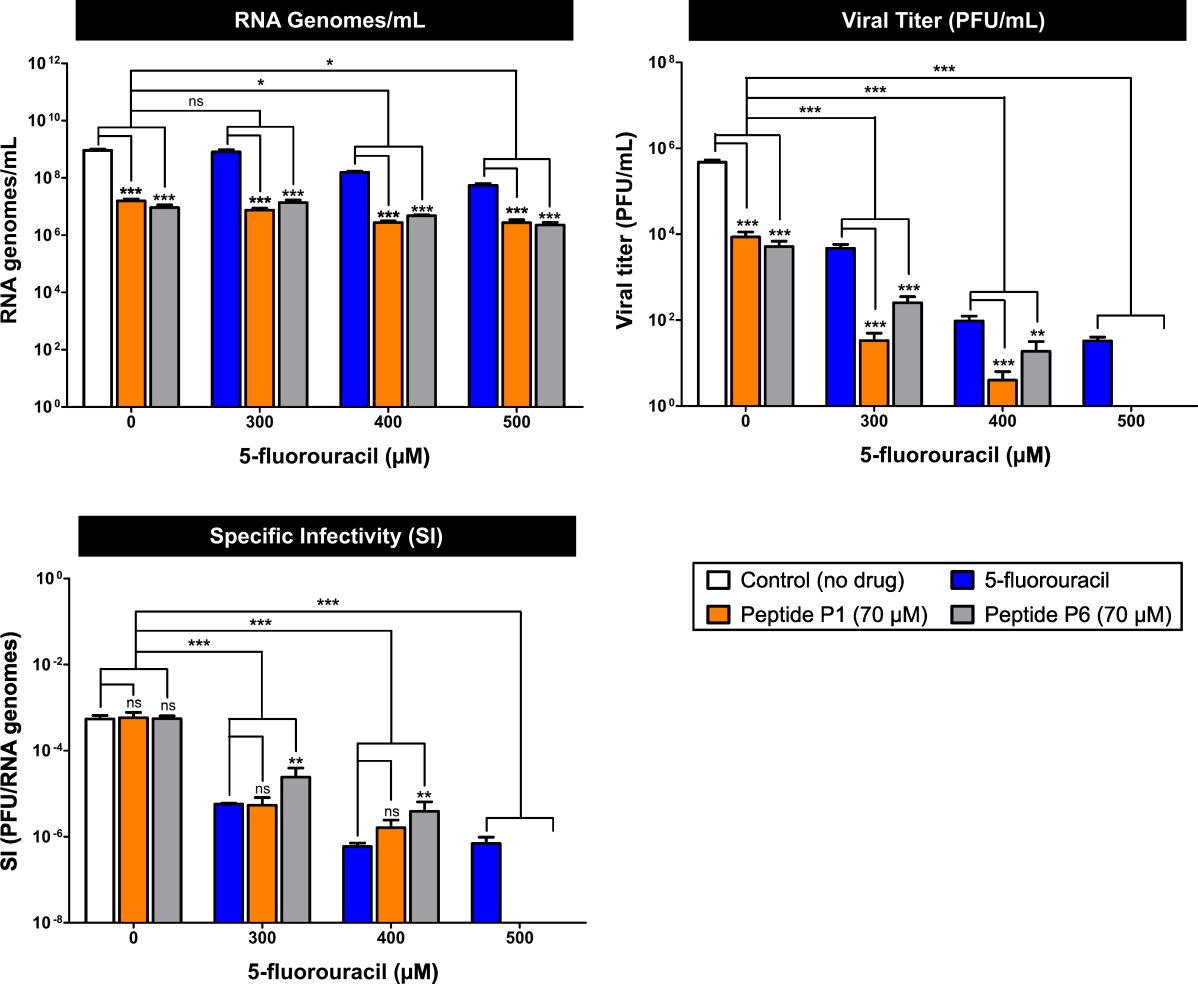
Effect of 5-FU and peptides P1 and P6 on the SI of SARS-CoV-2. Quantification of viral RNA copy number and viral infectivity (PFU/mL) under the indicated treatment conditions. Infectivity data, previously shown in Fig. 4, are presented here to provide an integrated overview of specific infectivity and mutant spectrum analyses. SI was calculated as the ratio of infectious units (PFU) to total viral RNA genomes. Bars represent mean ± SD (*n* = 3). Two types of statistical analyses were performed: (i) within each 5-FU concentration group (0–500 µM), comparing samples without peptide vs. samples treated with peptide; and (ii) between 5-FU-treated groups (300–500 µM) and the untreated control (X-axis = 0). White bars represent untreated controls. Statistical significance: *P < 0.05; **P < 0.01; ***P < 0.001; ns, not significant. Complete infectivity, RNA, and SI data are summarized in Table S4C.

5-FU monotherapy reduced viral titer by 10^2^-fold to 10^4^-fold, while RNA load remained relatively stable, with at most a ~10-fold reduction at 500 µM. As a result, SI decreased by ~100-fold (10^−4^–10^−6^ PFU/RNA genomes, Fig. 5; Table S4C), indicating that 5-FU primarily impaired infectivity without substantially suppressing viral RNA replication.

In contrast, peptides P1 and P6 reduced both viral titer and RNA load (~100-fold), leading to SI values similar to those of untreated controls (Fig. 5). This pattern suggests an inhibitory effect predominantly associated with viral replication.

Combined treatments produced complementary outcomes: peptides limited RNA genome accumulation, whereas 5-FU intensified loss of infectivity, particularly in combination with P1. As a consequence, SI values under combined treatments were markedly reduced and approached those observed for 5-FU monotherapy (Fig. 5; Table S4C). Overall, these results indicate that the combinations enhance infectivity inhibition while leaving RNA replication largely unaffected.

#### Mutation spectra and genetic diversity under drug exposure

The impact of antiviral treatments on both the number of mutations and the genetic diversity of mutant spectra was evaluated.

**Fig 6:**
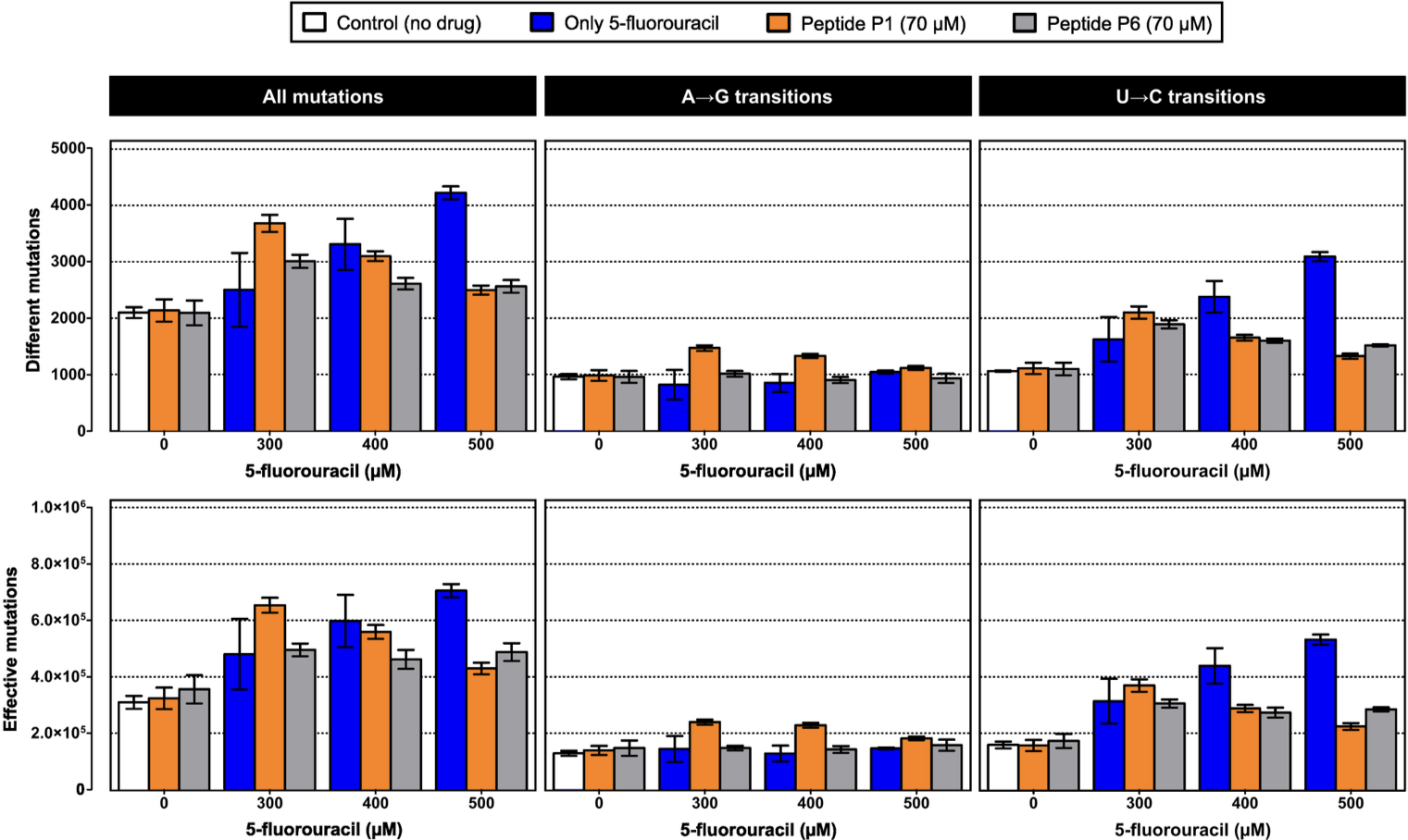
Mutation spectra of SARS-CoV-2 in the presence of 5-FU and peptides P1 and P6, alone or in combination. The plots show the number of different mutations (each counted once per sample) and the total number of effective mutations (each occurrence counted). The results are also shown separately for A→G and U→C transitions, which accounted for 75%–90% of all detected mutations. Bars are color-coded as in Fig. 4 and 5 (white, control; blue, 5-FU; orange, P1; gray, P6; mixed colors, combinations). Data represent mean ± SD (*n* = 3). Statistical comparisons are summarized in Table S5E. Complete lists of mutations and their frequencies are provided in Table S5B through D.

Consensus sequences were identical across all treatments (Fig. S2; Table S5A). Analysis of minority variation within the mutant spectra revealed that U→C and A→G transitions predominated under all conditions (Fig. 6; Table S5B and C). Most mutations appeared at very low frequencies (0.2%–1%), and only a small fraction exceeded 1% (Fig. S7).

**Fig 7:**
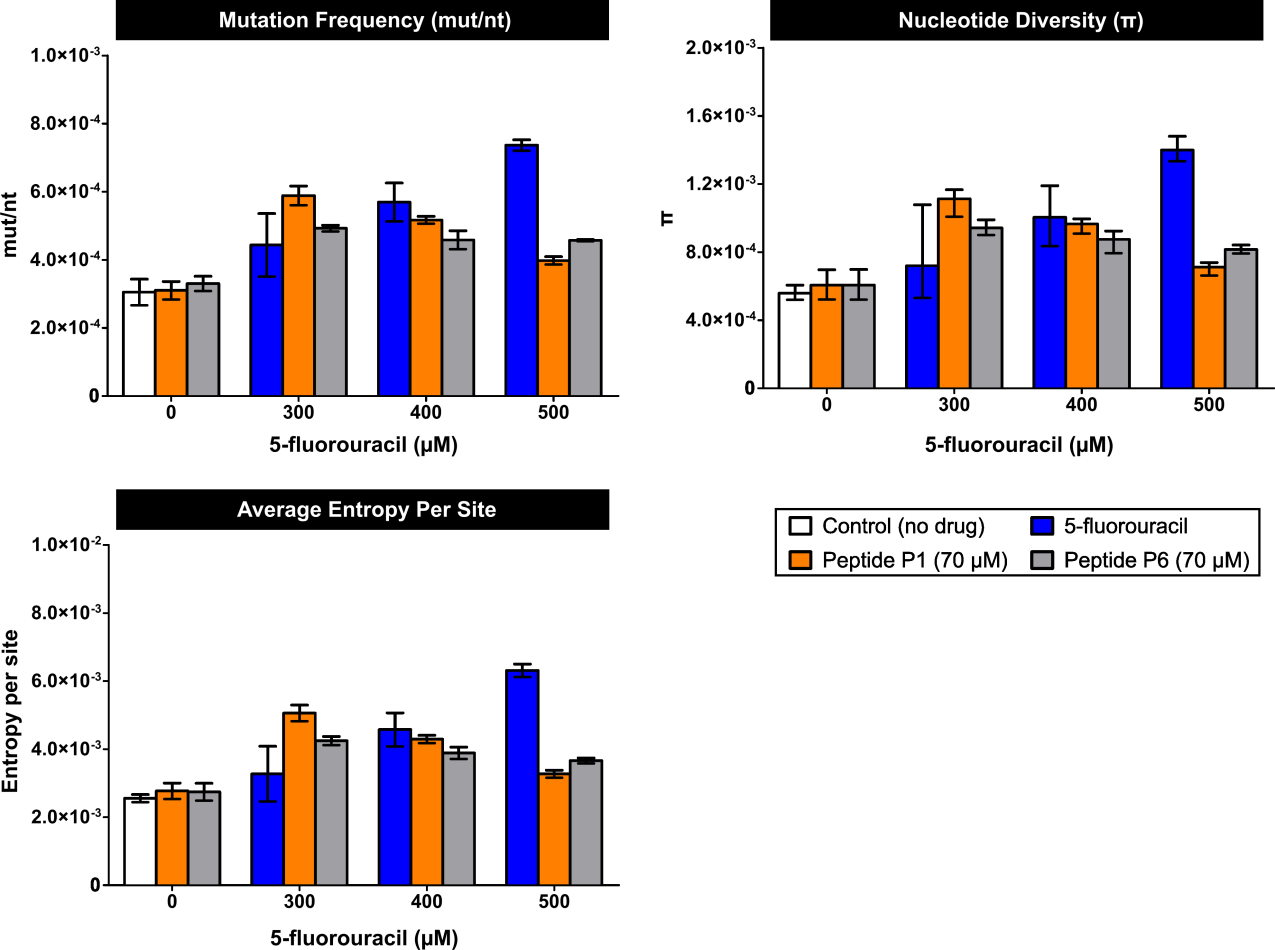
Genetic diversity of SARS-CoV-2 mutant spectra in the presence of 5-FU and peptides P1 and P6, alone or in combination. Diversity indices were calculated from NGS data and include mutation frequency, nucleotide diversity (π), and average Shannon entropy per site. Bars are color-coded as in Fig. 4 and 5 (white, control; blue, 5-FU; orange, P1; gray, P6; mixed colors combinations). Data represent mean ± SD (*n* = 3). Statistical analyses are provided in Table S5E, and complete diversity values in Table S5D.

5-FU treatment increased the number of mutations and the genetic diversity in a dose-dependent manner (Fig. 6 and 7; Table S5D), with statistically significant differences at 500 µM (Table S5E), confirming that high analog concentrations markedly alter the mutant spectra.

Peptides P1 and P6 alone did not significantly affect the number of mutations or diversity indices (Fig. 6 and 7; Table S5D). Their values remained significantly lower than those measured under all 5-FU treatments (Table S5E), indicating that the peptides do not exhibit mutagenic activity *per se*.

Combined treatments displayed a characteristic dose-dependent pattern. At low 5-FU concentrations (300 µM), the presence of peptides increased the number of mutations and diversity indices above those produced by the analog alone. As the 5-FU dose increased, the peptide co-treatment attenuated the diversity expansion normally induced by 5-FU. Thus, at 500 µM, the combinations generated significantly fewer mutations and lower diversity than 5-FU monotherapy, although still above untreated control levels (Fig. 6 and 7; Table S5D and E).

Distinct peptide-specific patterns were observed. In the 5-FU + P6 combination, mutation counts and diversity indices remained relatively stable across 5-FU doses. In contrast, the 5-FU + P1 combination showed a significant, dose-dependent decrease in genetic diversity (Fig. 6 and 7; Table S5D and E).

In addition, qualitative changes were observed in the distribution of mutations within the mutant spectra. The (A→G+U→C)/(G→A+C→U) ratio showed significant differences among treatments. As expected, 5-FU increased the frequency of U→C mutations, but A→G transitions remained similar to control groups. The analog also increased C→U and G→A transitions, thereby raising the Ts/Tv ratio (Tables S4C and 5D). Peptides P1 and P6 alone produced ratios comparable to the control. In contrast, the 5-FU + P1/6 combinations showed lower (A→G+U→C)/(G→A+C→U) and Ts/Tv ratios than 5-FU alone (Table S5D), largely due to reduced accumulation of U→C transitions (Fig. 6) at higher analog concentrations, together with a relative increase in transversions (Table S5C). This pattern suggests that peptides may modulate how replication errors induced by 5-FU are processed, limiting the accumulation of dominant 5-FU-driven mutations. These compositional changes are consistent with the dinucleotide deviations detected under combination treatments (Fig. 6), supporting the notion that peptide co-treatments reshape the balance of transition and transversion events and thereby modulate the mutational signature normally imposed by 5-FU.

We next examined whether drug treatments altered the genomic distribution of mutations. Runs test analysis (Table S5F) showed that in untreated controls, mutations were distributed non-randomly across the genome (*P* < 0.05), driven mainly by ORF1ab, whereas other ORFs displayed random distributions. Except for the 300 μM 5-FU condition, all treatments promoted a shift toward random distribution of mutations within ORF1ab, suggesting that exposure to antiviral agents modifies how mutations are distributed across the viral genome.

Dinucleotide composition analysis supported these observations. Control samples displayed enriched G/C-rich dinucleotides, including CpG, and reduced A/U-rich dinucleotides such as UpA, consistent with the predominance of U→C and A→G transitions. 5-FU alone did not significantly alter dinucleotide O/E ratios. In contrast, both peptide-only and combination treatments produced detectable deviations (Fig. S8; Table S5G), including further reductions in A/U-rich dinucleotides and enhanced shifts associated with CpG-related pattern, in agreement with the diversity modulation observed in the mutant spectra.

#### Multivariate analysis of mutant spectra

To integrate the effects of 5-FU and peptide treatments on the overall population structure, we performed a principal component analysis (PCA) based on mutational composition. The first two components explained 83.18% of the total variance.

**Fig 8:**
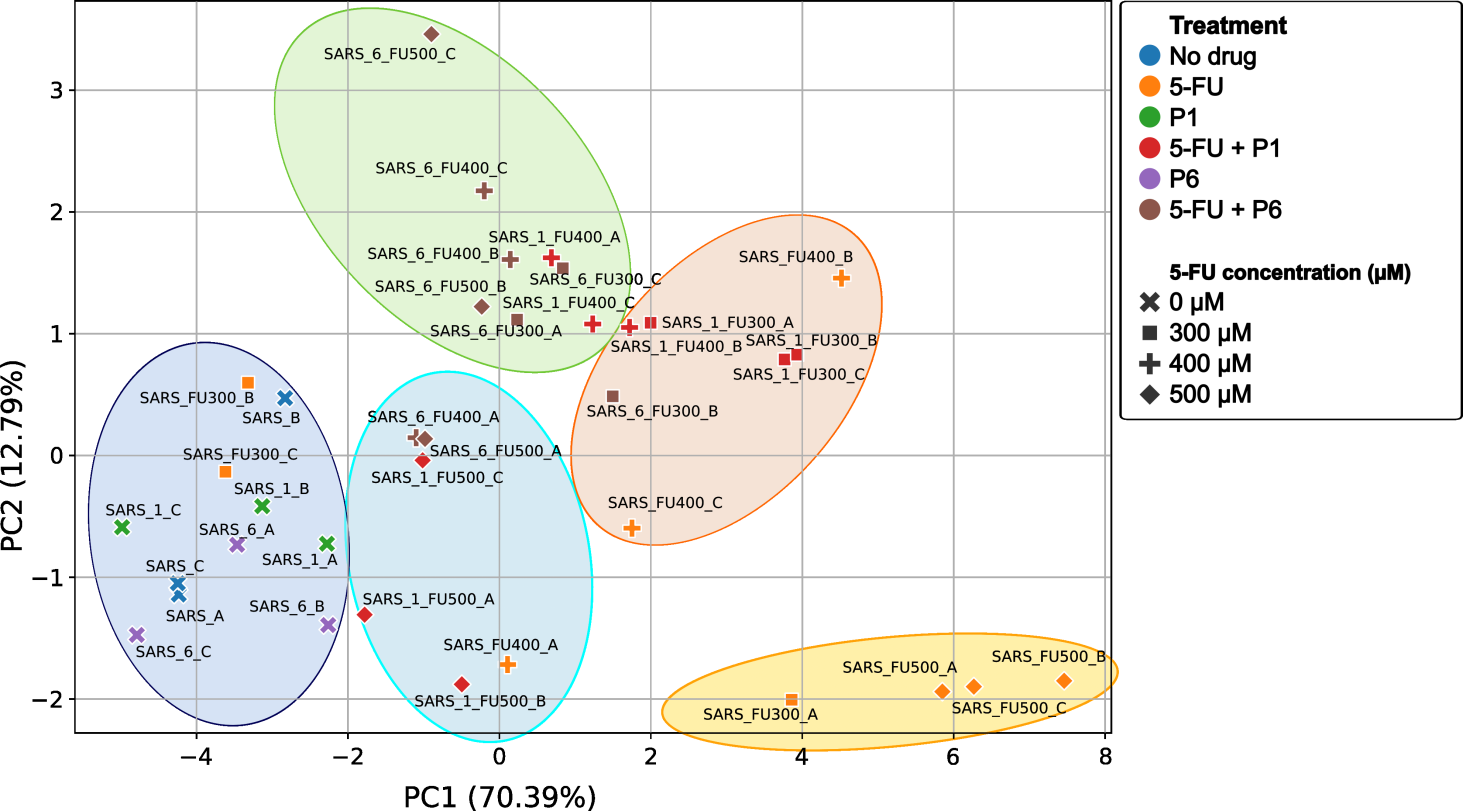
PCA of SARS-CoV-2 mutant spectra under different treatments. The scatter plot shows clustering of viral populations according to their mutational composition. The first two principal components explained 70.39% (PC1) and 12.79% (PC2) of the total variance. Controls (no drug, blue) and peptide-only treatments (P1, green; P6, magenta) formed tight clusters, whereas 5-FU monotherapy (orange) and combination treatments (5-FU + P1, red; 5-FU + P6, brown) showed distinct separation, depending on the analog concentration. Symbols indicate 5-FU doses: 0 µM (×), 300 µM (square), 400 µM (+), and 500 µM (diamond). Complete PCA parameters are provided in Table S5H, with additional visualizations in Fig. S9.

In the PCA plot (Fig. 8), untreated controls and peptide-only samples (P1 or P6) formed tight clusters, indicating highly homogeneous mutant spectra. Two of the three 300 µM 5-FU replicates (SARS_FU300_B and SARS_FU300_C) also clustered with controls, whereas the third (SARS_FU300_A) was clearly displaced, indicating heterogeneous responses at intermediate analog doses.

Combination treatments showed greater dispersion and clear separation from controls. Three well-defined clusters emerged, corresponding to treatment type and 5-FU concentration. A dose-dependent trend was evident: higher 5-FU concentrations progressively shifted samples further from the control/peptide clusters, indicating increasing divergence in mutational profiles.

Interestingly, the 5-FU + P1 combinations exhibited a modulatory effect. At 500 µM 5-FU, replicates clustered closer to controls and peptide-only treatments, suggesting that P1 attenuated the broad diversification induced by high-dose 5-FU. At 300 µM, however, P1 + 5-FU formed a distinct cluster shifted along PC2, separate from both controls and 5-FU alone. Combinations with P6 followed a similar but less pronounced pattern.

Finally, the 500 µM 5-FU monotherapy replicates, along with the divergent SARS_FU300_A sample, formed a compact cluster at the positive end of PC1, demonstrating that high-dose 5-FU was the dominant driver of quasispecies diversification, whereas peptides introduced secondary, concentration-dependent modulations.

## DISCUSSION

The decline in vaccine efficacy against emerging SARS-CoV-2 variants has underscored the need for complementary antiviral approaches capable of limiting viral replication and reducing immune escape. In this context, our study presents a dual antiviral approach that combines SARS-CoV-2-derived inhibitory peptides designed to target replication complex functions with lethal mutagenesis.

The pyrimidine analog 5-FU, widely used in chemotherapy for its inhibition of DNA synthesis (64), has also been shown to induce lethal mutagenesis in multiple RNA viruses by increasing mutation frequency and promoting the accumulation of deleterious mutations (18, 20, 47, 62). The peptides designed in this study are derived from nsp10, an essential cofactor of the coronavirus replication complex that activates the nsp14 exonuclease (ExoN) and stimulates the nsp16 2′-O-methyltransferase (2′-O-MTase) (34, 40, 45, 65–67).

Our computational predictions indicated that mutations increasing guanine and cytosine content impose the highest fitness penalties on SARS-CoV-2. This may help explain the discrepancy between the dominant mutations observed in mutant spectra (U→C and A→G) (68, 69) and the substitutions that become fixed in viral populations, where C→U mutations predominate (70, 71). Based on genomic dinucleotide frequencies, we identified which dinucleotides favor SARS-CoV-2 replication and confirmed the particularly detrimental effect of CpG, consistent with strong host immune pressure reported previously (72, 73). This rationale supported the selection of 5-FU, as it preferentially generates U→C and A→G transitions (18, 20, 62, 74). However, the fitness cost of specific substitutions differs across proteins, likely reflecting variation in structural stability (75) and protein-protein interfaces, where deleterious effects can be amplified (76). In nsp10, U→G and U→A were the most detrimental substitutions. Mutations that change uracil to any other nucleotide are especially detrimental for viral fitness, as they have the effect of reducing the hydrophobicity of the protein (77), since U in the second position almost always codes for hydrophobic amino acids. On the other hand, in the nsp10-nsp14 and nsp10-nsp16 complexes, G→U and C→U had the strongest predicted effects, consistent with their hydrophobic interfaces, where increased hydrophobicity can destabilize protein-protein interactions (78). These analyses highlight the limitations of prediction frameworks based solely on dinucleotide composition, as mutational impact also depends on the number, location, and structural consequences of substitution, at the nucleotide and protein levels, including effects on genomic features such as RNA secondary structures (79–81).

We did not test combinations of different peptides because several inhibitors were designed from the same nsp10 segment (residues 39–47: TNCVKMLCT), raising the possibility of competition for overlapping binding interfaces. Although each peptide was independently optimized for its target, interactions with shared nsp10 surfaces cannot be excluded (44, 54). Combining peptides could therefore hinder effective intracellular interactions by promoting competition, limiting antiviral potential (82–84). In addition, inhibition of the nsp10-nsp14 complex may affect not only ExoN but also N7-methyltransferase (N7-MTase) activity due to functional crosstalk between domains (44, 85) and could consequently impair mRNA capping and subsequent 2′-O-MTase function by nsp16.

The peptides exhibited markedly different IC_50_ and CC_50_ values despite high sequence similarity. For example, P1 and P2 differ by only two residues, yet displayed distinct cytotoxicity and antiviral activity. Even minor amino acid changes can strongly alter stability and interaction efficiency (86, 87). Native nsp10-derived peptide fragments were not evaluated experimentally, as the study focused on structure-guided optimized peptides with physicochemical properties compatible with cell-based antiviral assays. Peptide design required balancing hydrophobicity, necessary for membrane penetration and target binding (88), with solubility and low cytotoxicity (89, 90). Residues prone to chemical instability (e.g., Asp, which forms aspartimides) or redox reactivity (e.g., Cys) were avoided (91, 92). Cellular uptake also varied: P1 internalized more efficiently than P7, consistent with its higher positive charge, which promotes interactions with membrane proteoglycans and endocytic entry (93, 94). However, peptide uptake experiments were intended as a qualitative assessment of cellular association and fluorescence distribution. Surface-associated fluorescence cannot be fully excluded based on these images alone, and GFP alone was not included as a control in this assay. Importantly, peptide concentrations used in uptake experiments did not induce detectable cytotoxicity, arguing against major disruption of plasma membrane integrity. Although P7 showed antiviral activity, its evaluation was limited to internalization studies and low-dose combinations due to its narrower potency range relative to P1 and P6.

In our culture assays, 5-FU reduced SARS-CoV-2 titers by 100-fold to 10,000-fold at concentrations of 200–500 μM, among the strongest inhibitory effects reported for coronaviruses. In contrast, SARS-CoV exhibited only modest reductions (3-fold to 10-fold) at comparable concentrations (34), suggesting greater susceptibility of SARS-CoV-2 to nucleoside analogs, consistent with observations for β-D-N^4^-hydroxycytidine (NHC) (95–97). Differences in proofreading efficiency (98, 99) and the slightly higher mutation rate of SARS-CoV-2 (100) compared with SARS-CoV (101) may contribute to this increased susceptibility.

The combination of 5-FU with peptides P1 or P6 produced a markedly synergistic antiviral effect, most evident in infectivity assays. Peptide monotherapy reduced both infectious titers and RNA levels, and the addition of 500 μM 5-FU eliminated detectable infectivity, an outcome not achievable with 5-FU alone at non-cytotoxic concentrations. Synergistic effects typically emerge when drugs target complementary replication processes (102–105), as observed with combinations such as molnupiravir plus TMPRSS2 inhibitors (106) or ribavirin plus remdesivir, which achieved viral extinction in culture (23). Such combinations improve efficacy and reduce toxicity by lowering required doses (107–109). When ribavirin is combined with remdesivir (23) or nirmatrelvir (110), lower concentrations of each drug are needed to inhibit SARS-CoV-2 replication.

Analysis of mutant spectra confirmed that 5-FU primarily reduced infectivity without strongly affecting genome replication, a hallmark of lethal defection (18, 111–113). Peptides alone exhibited only weak mutagenic activity, which may be consistent with partial interference with nsp14-dependent proofreading (34, 66, 114). Their modest effects on diversity may reflect limitations in intracellular stability and access. Unlike nucleoside analogs, peptides face barriers including protease degradation, low membrane permeability, rapid clearance, and potential immunogenicity (115–117). Additionally, SARS-CoV-2’s high mutation rate could generate variants with altered peptide sensitivity, even without direct mutations at binding sites (118, 119). Interference with nsp16 methyltransferase activity may also contribute to antiviral effects, as disruption of 5′-cap formation can impair viral RNA synthesis (120–122). Indeed, peptide treatments caused a substantial reduction in viral RNA relative to controls and 5-FU alone, suggesting that decreased replication could counterbalance mutational accumulation.

Patterns of genetic diversity under combination treatments provided additional insight into viral evolutionary responses to drug pressure. Dual treatment reduced genetic diversity in mutant spectra relative to 5-FU alone, especially at 500 μM, possibly due to peptide-induced suppression of replication rounds. Viral replication rate influences analog incorporation and reduced replication can limit mutagenesis (123–127). A reduction in replication may favor extinction, as replicative fitness itself can act as a resistance mechanism (128–130). However, not all analog combinations produce this effect, for example, ribavirin + remdesivir (23) and molnupiravir + nirmatrelvir (131).

The relationship between replication rate and analog-induced mutagenesis does not fully explain the qualitative changes in mutational spectra. If lower diversity were solely due to reduced replication rounds, all transitions would decrease proportionally. Instead, combination treatments produced decreases in the (A→G+U→C)/(G→A+C→U) ratio and Ts/Tv values, driven by a preferential reduction of U→C-5-FU’s signature mutation, without comparable decreases in other transitions. This suggests that peptides P1 and P6 may interfere with 5-FU’s mutagenic activity.

An intriguing dose-response pattern emerged at 300 µM 5-FU; combination treatments increased genetic diversity relative to 5-FU alone, despite viral load decreasing ~100-fold. This indicates that reduced replication rounds did not limit 5-FU-induced mutagenesis at this dose and may reflect a phase of lethal defection, where specific infectivity falls while mutation counts rise (18, 111). However, at higher 5-FU concentrations, diversity decreased under dual treatment relative to monotherapy, particularly with P1, although viral load remained stable. These findings suggest that peptides modulate the mutagenic response to 5-FU, enhancing mutagenesis at moderate doses but dampening it at high doses. Whether this behavior reflects changes in 5-FU incorporation efficiency (as FUTP) (21, 132, 133) or differences in 5-FU metabolism remains unknown and warrants further study through quantification of 5-FU metabolites and targeted proteomics.

Peptide-mediated interference with nsp14- and nsp16-associated functions may also affect post-replicative RNA maturation and innate immune evasion. These enzymes are essential for 5′-cap formation, which masks viral RNA from host sensors (134, 135). A previous study showed that an nsp16-targeting peptide (TP29) enhanced IFN-I production in mouse cells (46). This mechanism could not be evaluated in Vero E6 cells due to their lack of IFN-α/β genes (136). Although these cells can retain partial responses via IFN-λ downstream signaling pathways (RLR, Jak-STAT, TLR, and MAPK), immunomodulatory effects of peptide inhibition are likely underestimated (137, 138). Furthermore, differences in cytopathic effects between Vero E6 and human cells, particularly for Omicron variants, highlight the need for testing in immune-competent, physiologically relevant models (139). Therefore, confirmation of these antiviral effects in additional human respiratory cell systems will be an important objective for future studies. Moreover, changes in dinucleotide composition observed under peptide + 5 FU treatments reinforce this interpretation. CpG and UpA suppression are deeply conserved features of coronavirus genomes linked to innate immune evasion and RNA stability (30–32, 140), and even moderate deviations from these patterns can indicate perturbations of population robustness and genome architecture. The shifts detected in our combined treatment, therefore, suggest that dual therapy not only alters mutation frequency and diversity but also modulates structural constraints on genome composition, potentially reshaping the mutational landscape that sustains SARS-CoV-2 quasispecies dynamics. Such compositional deviations further support the view that quasispecies dynamics, rather than isolated mutations, underline the response of SARS-CoV-2 populations to combined mutagenic and peptide-based inhibition.

Although direct peptide-protein binding was not experimentally assessed in this study, the structure-guided design, conservation of the targeted interfaces, and the observed antiviral and synergistic effects are consistent with functional interference at nsp10-associated replication complexes, although additional biochemical studies will be required to fully define the underlying molecular interactions.

In conclusion, combining 5-fluorouracil with nsp10-derived peptides predicted to interfere with nsp14- and nsp16-associated functions produces a potent synergistic antiviral effect, markedly reducing SARS-CoV-2 infectivity. This dual mechanism, acting on proofreading- and methylation-associated processes while enhancing mutagenesis, supports the promise of peptide-nucleoside analog combinations as a broad-spectrum antiviral strategy. However, the behavior of mutant spectra under dual treatment remains complex and requires additional studies to clarify underlying mechanisms. Future work in immune-competent systems and across diverse viral variants will be crucial to validate the efficacy, safety, and translational potential of this approach as a potential pan-coronavirus therapeutic platform.

## Data Availability

The data generated and analyzed in the present study are included in this manuscript and the supplementary material. Additional information is available from the corresponding author upon reasonable request. Illumina raw read data has been uploaded to the Sequence Read Archive (SRA) database under accession ID PRJNA1379670.

## SUPPLEMENTAL MATERIAL

Supplemental figures aac.01885-25-s0001.docx Fig. S1 to S9.

Table S1 aac.01885-25-s0002.xlsx Materials and methods.

Table S2 aac.01885-25-s0003.xlsx Estimated effects of individual mutations on SARS-CoV-2 fitness.

Table S3 aac.01885-25-s0004.xlsx Evolutionary analysis of coronavirus proteins.

Table S4 aac.01885-25-s0005.xlsx Cell culture assays.

Table S5 aac.01885-25-s0006.xlsx Quasispecies analysis.

ASM does not own the copyrights to Supplemental Material that may be linked to, or accessed through, an article. The authors have granted ASM a non-exclusive, world-wide license to publish the Supplemental Material files. Please contact the corresponding author directly for reuse.
