# Supplementary material for: Synergistic antiviral effects of structure-guided peptides and a mutagenic base analog on SARS-CoV-2 replication
Source: Antimicrob Agents Chemother. 2026 Apr 27;70(6):e01885-25. doi: 10.1128/aac.01885-25 (PMC13231887; doi:10.1128/aac.01885-25)
Supplement: Supplemental figures — Fig. S1 to S9. [file aac.01885-25-s0001.docx]

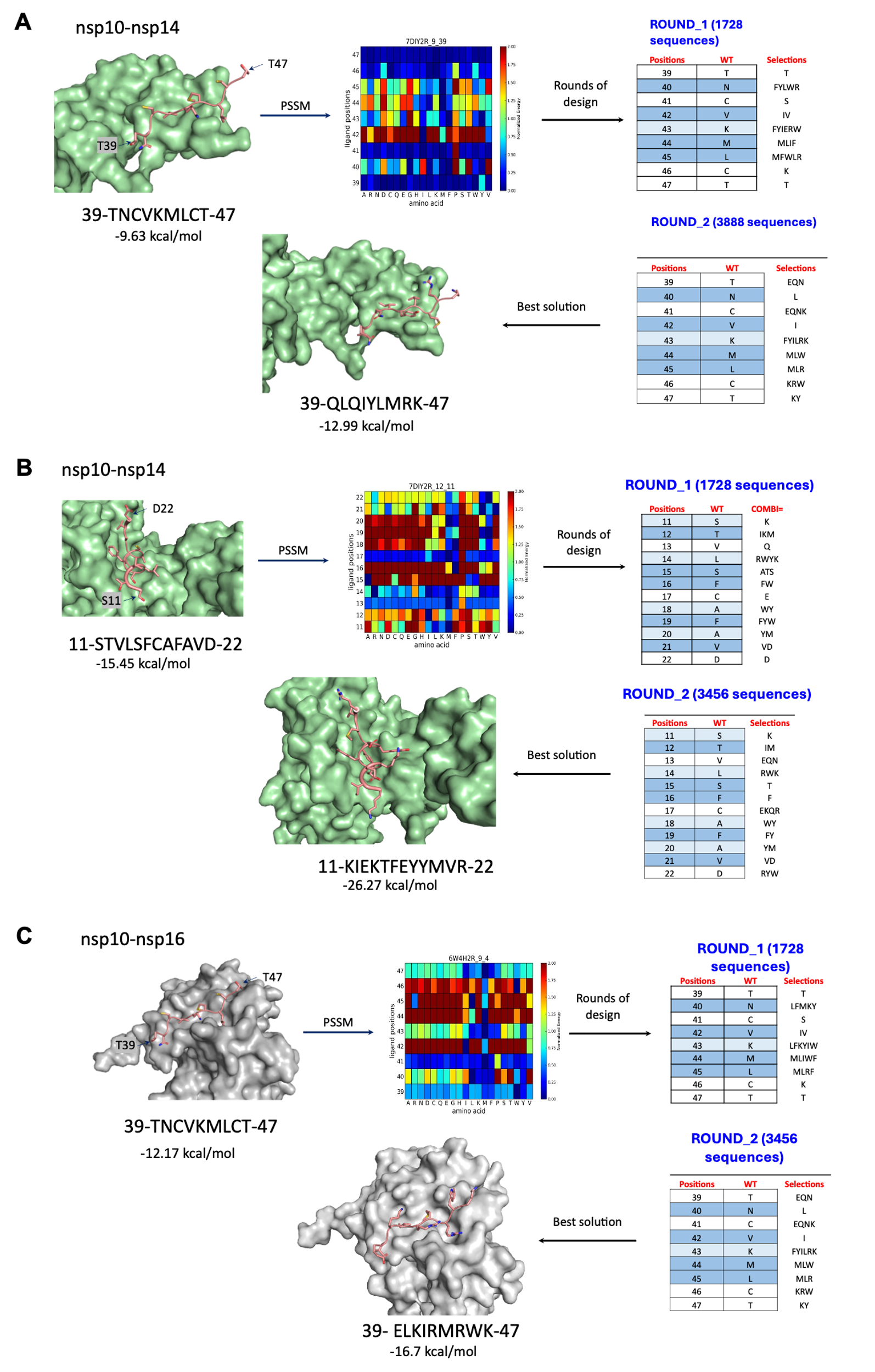


**Figure S1.** **Sequence space explored at each position of the selected peptides within their structural environment.** Position-specific scoring matrices were calculated using protein–peptide complexes derived from natural interactions. Two design rounds were performed: the first explored buried and semi-buried positions to reduce the number of complexes to model, and the second explored exposed positions and re-evaluated key sites from round 1. A set of optimal peptides was obtained and ranked according to interaction energy. (A) nsp10 peptide 39–47 (salmon) bound to nsp14 (green surface); (B) nsp10 peptide 11–22 (salmon) bound to nsp14 (green surface); (C) nsp10 peptide 39–47 (salmon) bound to nsp16 (gray surface).


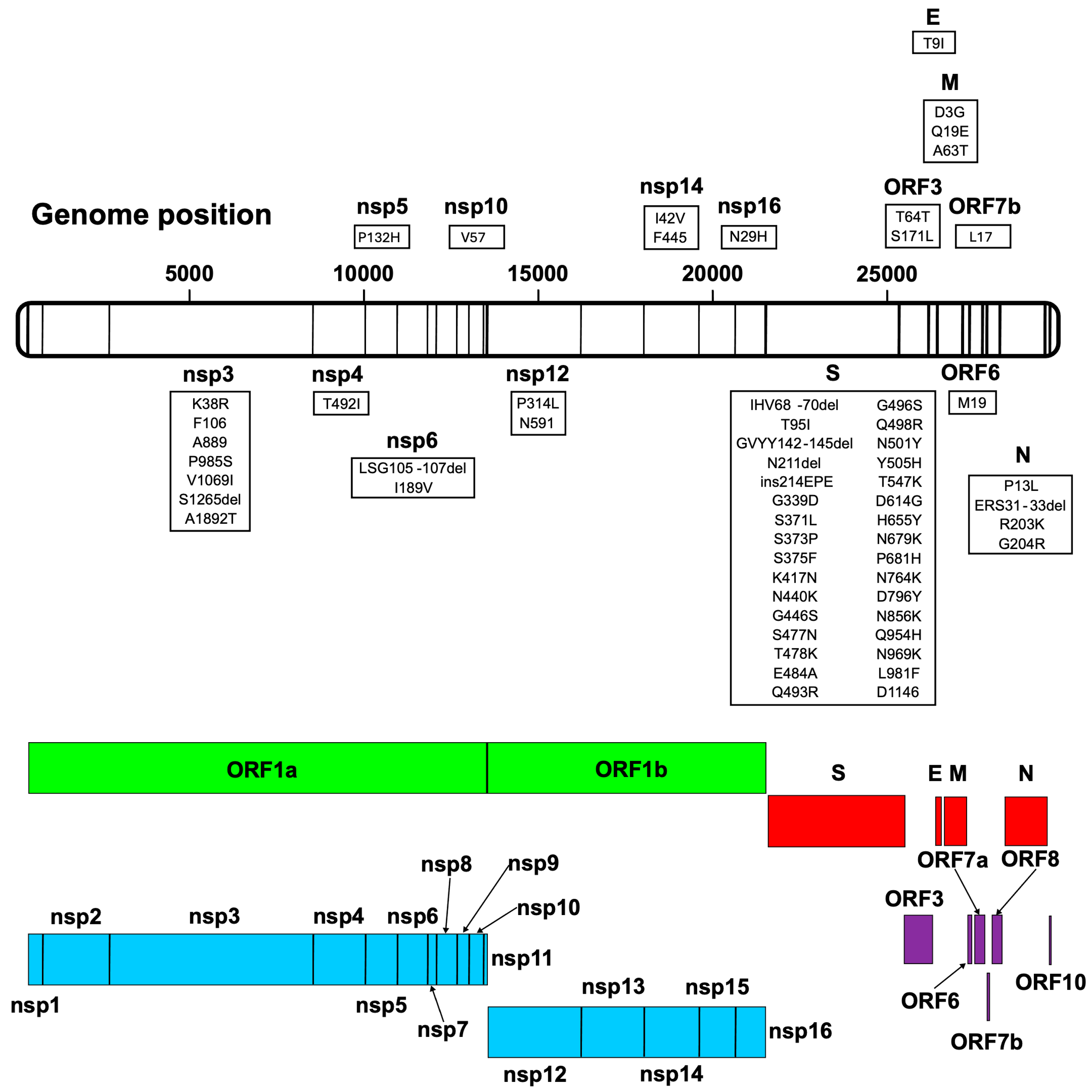


**Figure S2. Consensus sequence analysis of SARS-CoV-2 samples used for bioinformatic studies.** Consensus sequences obtained under each experimental condition were identical to that of the viral stock (passage 3). The schematic representation shows the location of open reading frames (ORFs) and the proteins encoded by each ORF. Detected mutations are shown in boxes corresponding to each protein. For synonymous substitutions, only the amino acid position is indicated (e.g., V57). The Wuhan-Hu-1 sequence (GenBank accession no. NC_045512.2) was used as the reference. Mutations fixed in consensus sequences are summarized in Table S5A. *Abbreviations: ins, insertion; del, deletion*.


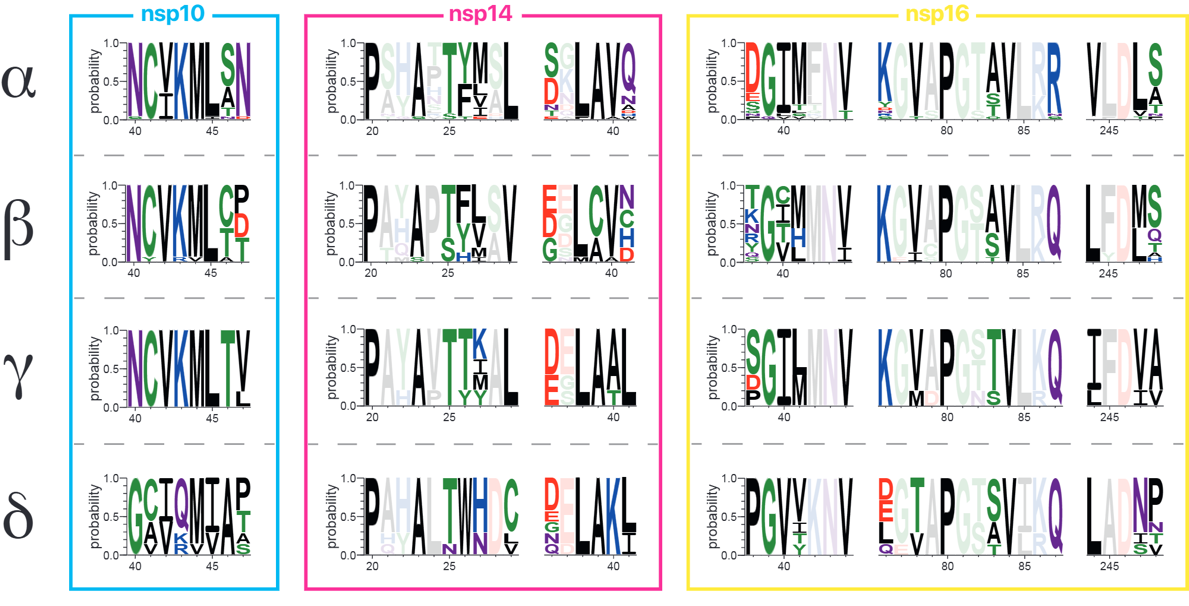


**Figure S3. Logos of amino acid sequences from the nsp10 domain (cyan) selected in this study and the regions of nsp14 (magenta) and nsp16 (yellow), relating to the genera *Alphacoronavirus* (α), *Betacoronavirus* (β), *Gammacoronavirus* (γ), and *Deltacoronavirus* (δ).** Residues are shown in different colors: hydrophobic = black, polar = green, basic = blue, acidic = red, neutral = purple. The positions of the residues indicated on the X-axis correspond to those occupied in the reference structures of nsp10/nsp14 ExoN (PDB ID: 7DIY) and nsp10/nsp16 (PDB ID: 6W4H) used in this work. The weakly colored positions of nsp14 and nsp16 do not participate in direct interaction with nsp10 residues.


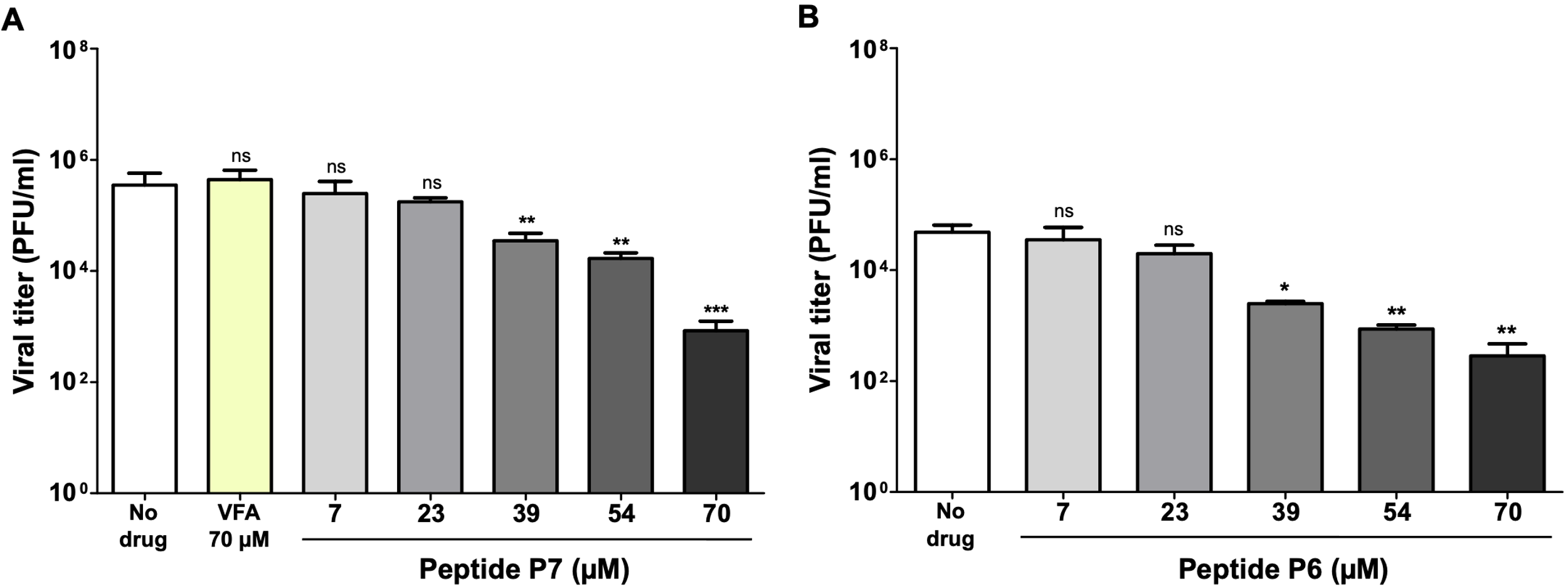


**Figure S4. Infectivity of SARS-CoV-2 in Vero E6 cells after treatment with peptides P7 (A) and P6 (B).**Vero E6 cells were infected with SARS-CoV-2 and treated with increasing concentrations of peptides P7 or P6 (7–70 µM). Viral titers (PFU/mL) were determined 48 h post-infection by plaque assay. A peptide with antiviral activity specific for foot-and-mouth disease virus (VFA, 70 µM) was used as control (provided by Dr. Francisco Sobrino, CBMSO). Bars represent mean ± SD (n = 3). Asterisks indicate significant differences within experimental groups (*p < 0.05; **p < 0.01; ***p < 0.001); ns = not significant.


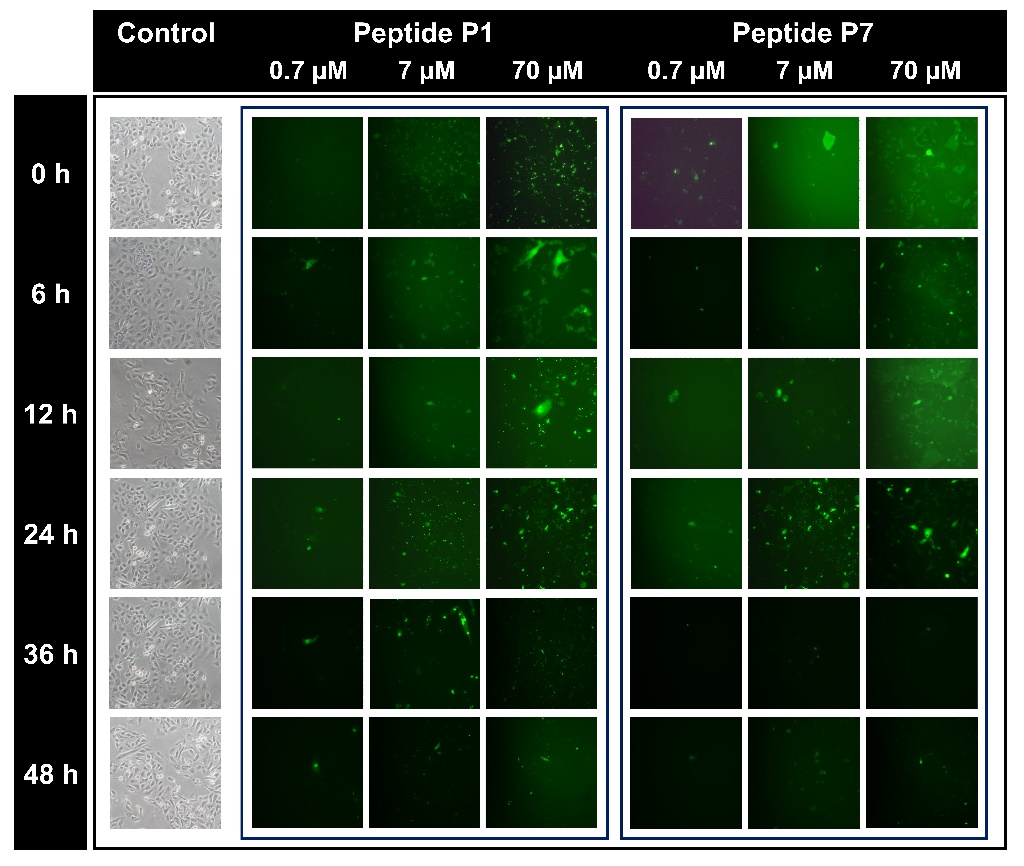


**Figure S5. Cellular association of GFP-labeled peptides P1 and P6 in Vero E6 cells.**  Vero E6 cells were incubated with GFP-labeled peptides P1 and P7 at 0.7, 7, and 70 µM, and fluorescence was monitored by microscopy at 0, 6, 12, 24, 36, and 48 h post-treatment. Bright-field images correspond to untreated controls. Both peptides showed time- and concentration-dependent fluorescence signals consistent with cellular association and uptake, with P1 producing stronger and more homogeneous fluorescence signals than P7. At the lowest concentration (0.7 µM), fluorescence was faint or absent, whereas signal intensity decreased after 36 h, consistent with partial peptide degradation or turnover. These experiments provide a qualitative assessment of peptide cellular association; surface-bound fluorescence cannot be fully excluded based on these images alone.

**
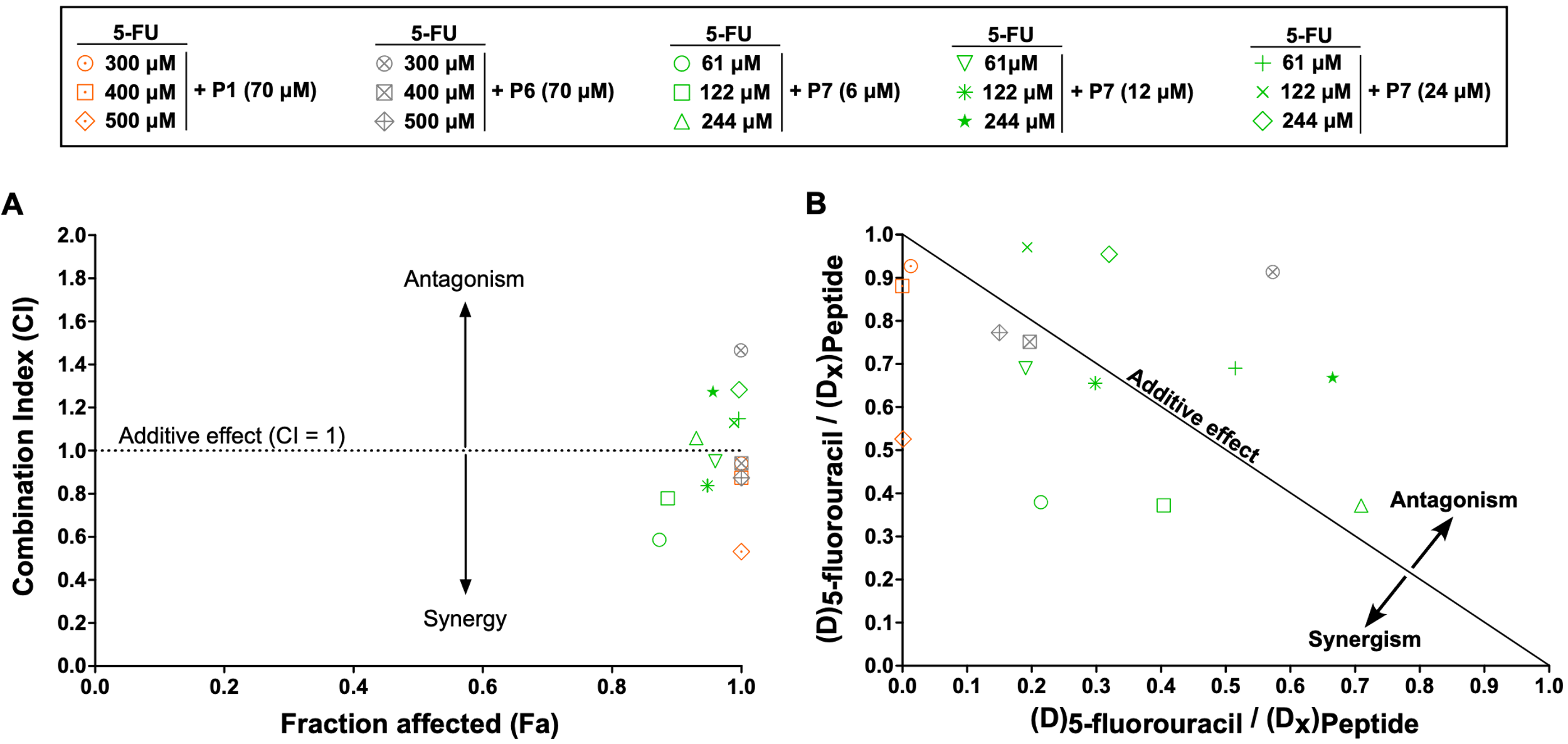
**

**Figure S6. Synergistic activity of 5-fluorouracil (5-FU) in combination with selected peptides against SARS-CoV-2.** Drug interactions were analyzed using the Chou-Talalay method in *CompuSyn*. (A) Combination Index (CI) values for 5-FU and peptides as a function of the fraction affected (Fa). CI values <1, =1, and >1 indicate synergy, additivity, and antagonism, respectively. (B) Dose-normalized isobologram for the forward combinations of 5-FU with peptides P1, P6 and P7. Data points on the hypotenuse indicate additivity; points in the lower-left triangle indicate synergism; points in the upper-right triangle indicate antagonism. The symbol code for drug concentrations is shown in the box above the plot.

**
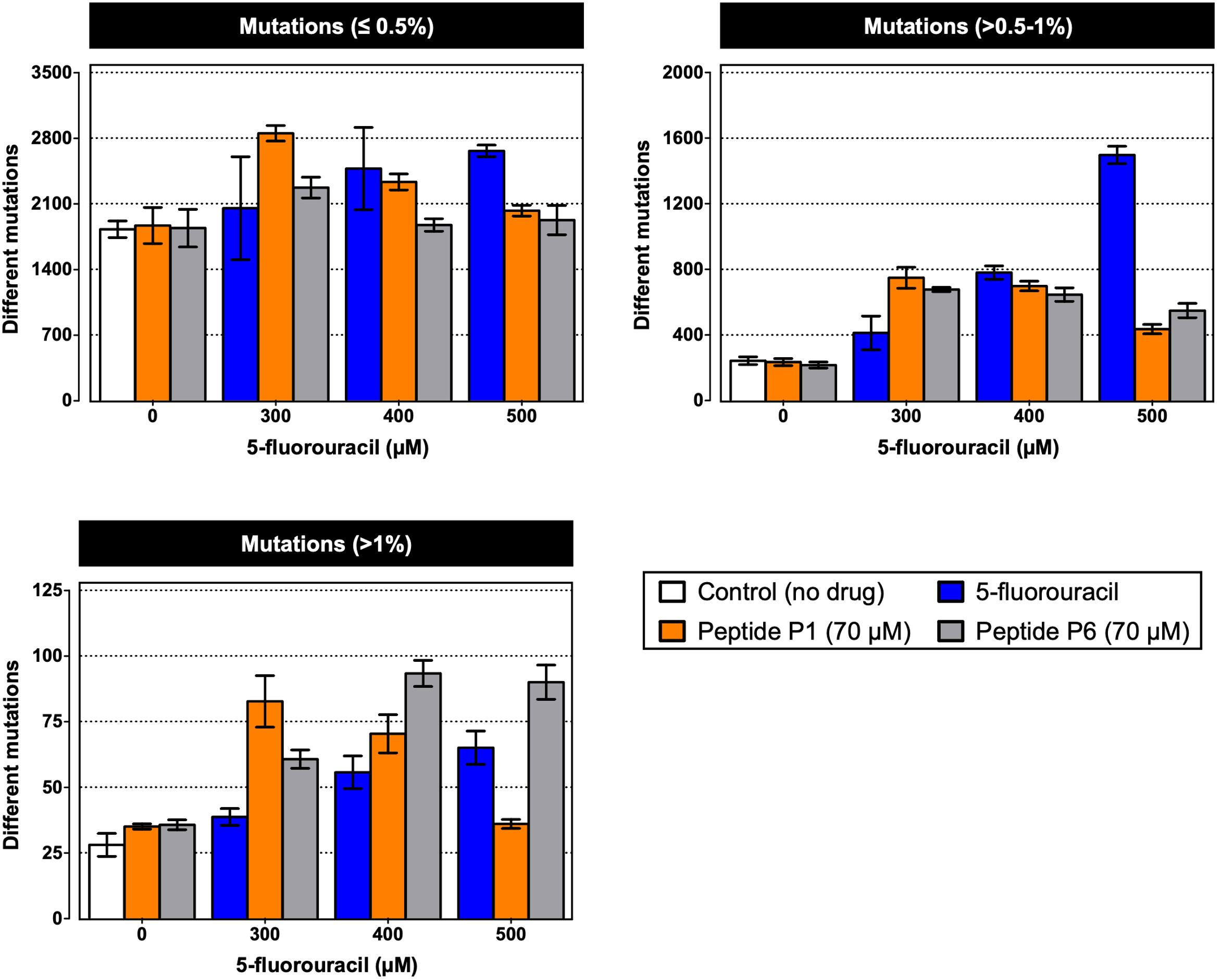
**

**Figure S7. Frequency distribution of point mutations in SARS-CoV-2 mutant spectra under different treatments.** Mutations detected in viral populations cultured in the absence or presence of 5-fluorouracil (5-FU), peptide P1, peptide P6, or their combinations were grouped according to frequency ranges (≤ 0.5%, > 0.5–1%, > 1%). White bars correspond to untreated controls. Data represent mean ± SD (n = 3). Complete information on detected mutations is provided in Tables S5B–D.

**
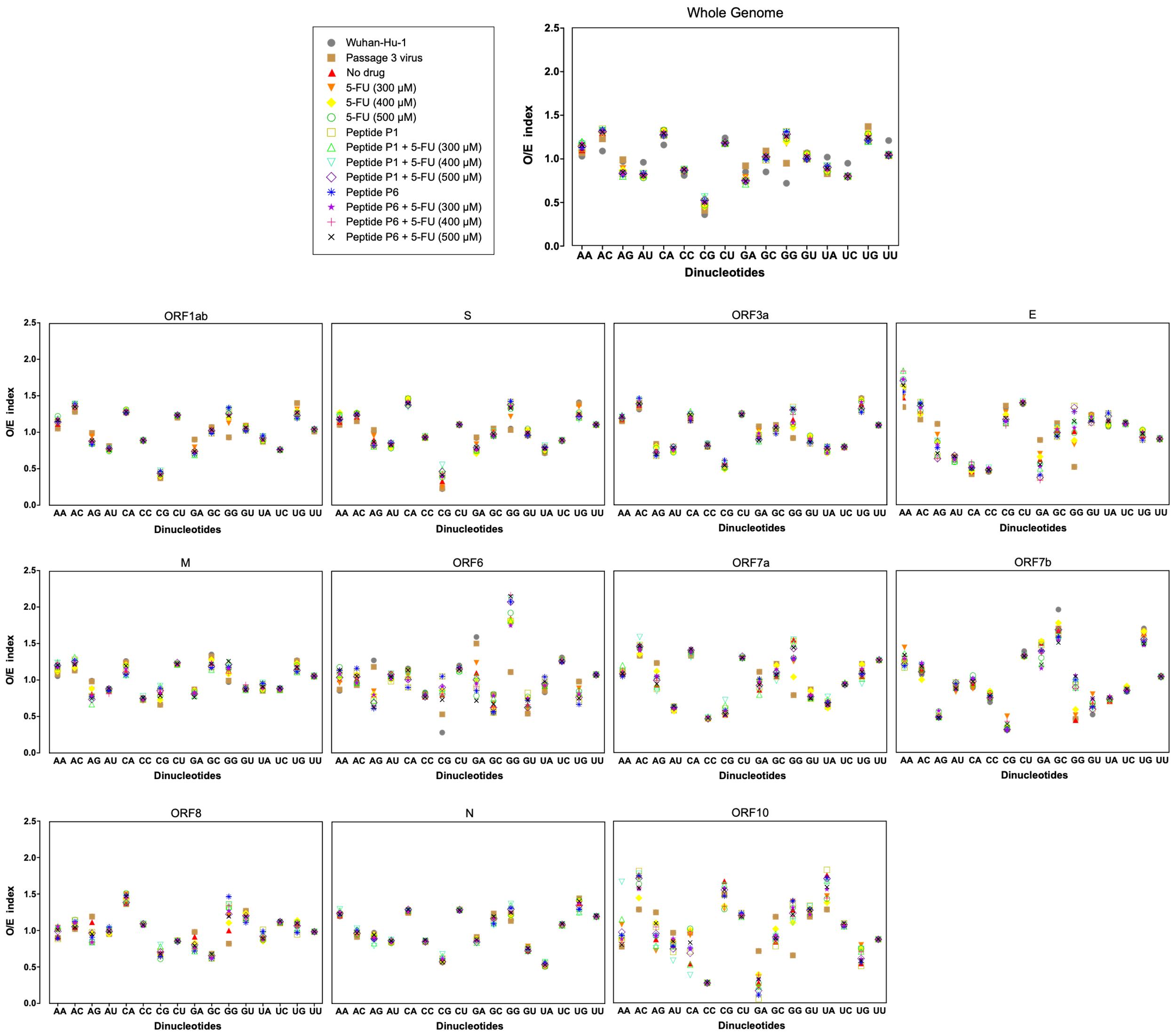
**

**Figure S8. Observed-to-expected (O/E) ratios of dinucleotide frequencies in SARS-CoV-2 mutant spectra.** Values were calculated at both the whole-genome and individual ORF levels. Results are shown for the untreated control, 5-fluorouracil (5-FU), peptides P1 and P6, and their combinations. Reference values from the Wuhan-Hu-1 sequence (GenBank accession no. NC_045512.2) and from the viral stock (passage 3) are also included (GenBank accession no. PV483427.1). Complete O/E ratio data for each condition are summarized in Table S5G.

**
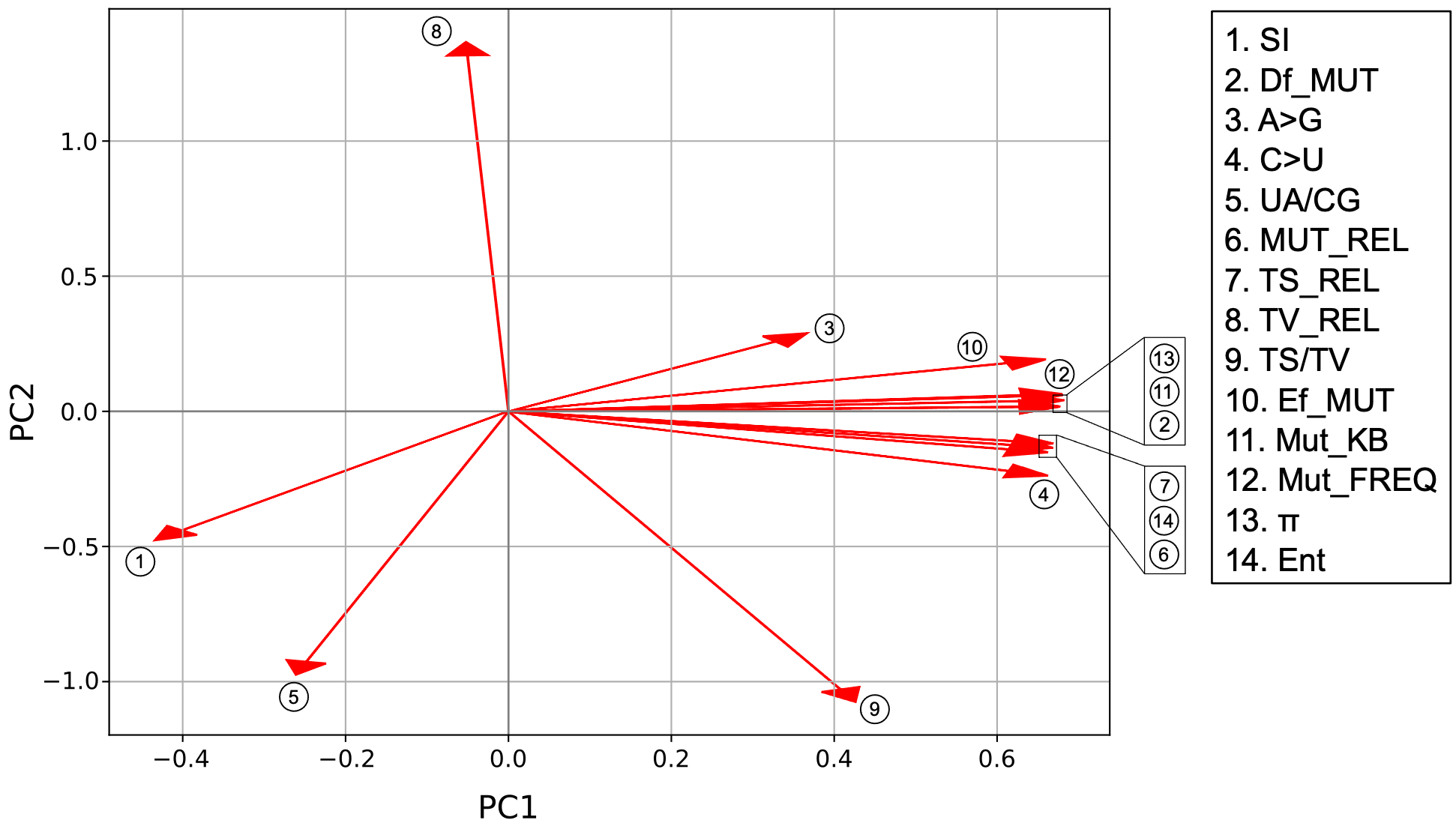
**

**Figure S9. Variable distribution map defining the two-dimensional PCA of SARS-CoV-2 mutant spectra.** The names of the main components are abbreviated. The names of each parameter and their definitions are compiled in Table S5H.
